# Dedifferentiated Schwann cell-derived TGF-β3 is essential for the neural system to promote wound healing

**DOI:** 10.7150/thno.72317

**Published:** 2022-07-18

**Authors:** Min-Yi Ou, Poh-Ching Tan, Yun Xie, Kai Liu, Yi-Ming Gao, Xiao-Sheng Yang, Shuang-Bai Zhou, Qing-Feng Li

**Affiliations:** 1 Department of Plastic & Reconstructive Surgery, Shanghai Ninth People's Hospital, Shanghai Jiao Tong University School of Medicine, Shanghai 200011, China.; 2 Department of Neurosurgery, Shanghai Ninth People's Hospital, Shanghai Jiao Tong University School of Medicine, Shanghai 200011, China.

**Keywords:** Single-cell transcriptome, skin, Schwann cells, wound repair, TGF-β

## Abstract

**Rationale:** Wound healing is among the most complicated physiological processes and requires the synchronization of various cell types with distinct roles to re-establish the condition of the original skin. Patients affected by peripheral neuropathies often experience failure to heal. Loss of Schwann cells (SCs), a crucial population of peripheral nervous system cells in skin, may contribute to chronic wounds. However, the role of SCs in wound healing are poorly understood.

**Methods:** The activity of SCs was investigated by using a cell atlas of the wound healing process, which was generated by integrating single-cell RNA sequencing (scRNA-seq) libraries covering different states of mouse back skin. The results of in silico analysis were validated by *in vitro* cell culture and *in vivo* mouse model. Selective inhibitors and conditional RNAi by virus transfection were utilized to investigate the role of SCs in wound healing. Findings from mouse experiments were further verified in scRNA-seq analysis of diabetic patients.

**Results:** Our in silico analysis revealed the heterogeneous cellular components of skin and the dynamic interactions of neural crest derived cells (NCs) with other cell types. We found that SCs dedifferentiated at an early stage of wound repair with upregulated Wnt signaling. We also identified dedifferentiated SC (dSC) defect in diabetic wounds in both mouse and human. Wnt inhibition at the wound site repressed SC dedifferentiation, leading to defective repair. Furthermore, dSCs derived TGF-β3, which is context-dependent, promoted the migration of fibroblasts and keratinocytes. Moreover, TGF-β3 supplementation enhanced the healing of chronic wounds in diabetic mice with impaired SCs.

**Conclusion:** Our study thus advances the understanding of the roles of neural-derived cells in skin regeneration and suggests a potential therapeutic strategy for wound healing disorders.

## Introduction

The skin, the key boundary of the human body that protects internal organs from various types of damage, is the most frequently injured tissue. Millions of people worldwide are afflicted with chronic nonhealing wounds every year, which imposes a substantial economic burden on the public and individuals. Wound healing is a complicated regeneration process that requires the coordination of multiple cell types at precise steps to operate repair program 1. The lack of knowledge about the roles of each cell type involved and the underlying mechanisms impedes the development of effective wound healing therapies 2. An increasing number of studies support that the nervous system is critical for multiple tissue regeneration processes, including wound healing. Traumatic or pathologic malfunction of the nervous system impacts wound healing 3-5. Chronic wounds are common in diabetic patients with dysregulated peripheral nervous systems. Obviously reduced innervation and decreased expression of neurogenic factors are observed in the foot ulcers of patients with both neuropathic and nonneuropathic diabetes and are correlated with inflammatory accumulation 6. Interestingly, Topical application of Substance P (SP), a neuropeptide from the nervous system, significantly accelerates cutaneous wound healing and is suggested to be a promising treatment for chronic diabetic wounds 7.However, how neural-derived cells participate in skin repair is not entirely clear.

In addition to the function of innervation, glial cells that modulate and support nerve activity have broader roles in tissue homeostasis and regeneration 8. Schwann cells (SCs), which are glial cells in the peripheral nervous system, have been extensively investigated in the context of nerve repair and are recognized as promising targets for nerve injury therapy. Dedifferentiated SCs (dSCs) are activated immediately after nerve damage and initiate a series of wound responses, including dedifferentiation, secretion of cytokines/chemokines and growth factors, phagocytosis of cellular debris and extracellular remodeling, which creates an appropriate microenvironment to support axon regrowth 9. Previous study revealed that dSCs play an essential paracrine role in adult dermal regeneration 10, 11. However, how SCs dedifferentiate following skin lesions and the molecular mechanism mediating SC regulation of wound healing in skin remain unclear. Besides, the relationship between SCs and regeneration defect in diabetic patients with neuropathy has not been addressed in previous study.

It may be difficult to cover all populations of SCs with traditional methods such as immunostaining, which relies on known signatures to label specific cells and is limited by the time point of sampling. The use of time-consuming FACS to enrich SCs may not ideally illustrate true activity and communication with surrounding cells. Single-cell RNA sequencing (scRNA-seq), which can identify all cell types in specific tissues with a relatively fast and simple sample preparation process, is a promising tool for deciphering cellular alterations and communications of SCs in specific circumstances.

To elucidate the role of neural cells in cutaneous wound healing, we analyzed scRNA-seq libraries to identify neural cells and found that SC dedifferentiation occurred at an early stage of the healing process with high Wnt signaling. Surprisingly, dSCs defect existed in diabetic wounds of both mouse and human. Using Wnt inhibition at wound sites *in vivo*, we found that Wnt signaling activation was involved in the dedifferentiation of SCs. By modulating TGF-β signaling *in vitro* and *in vivo*, we found that TGF-β3 mediated dSC activity, promoting wound healing at an early stage. Finally, we demonstrated that administration of TGF-β3 in injured skin improved chronic wounds in diabetic mice. Our findings led to the discernment of SC function in skin repair and the possibility of leveraging dSCs in potential therapy of chronic wounds.

## Results

### Single-cell transcriptomic analysis of small wounds reveals different skin cell types and their communications

**Figure 1:**
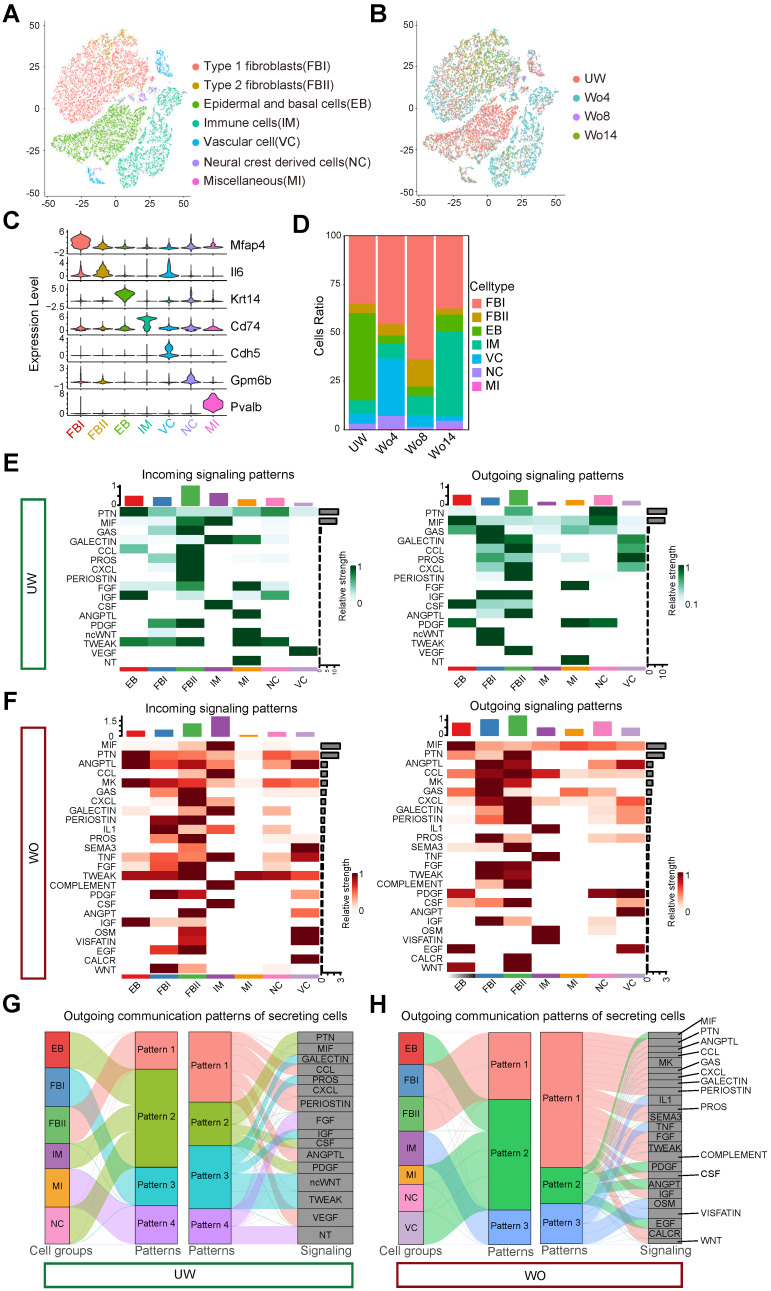
** Single-cell analysis of mouse skin before and after injury. (A)** T-distributed stochastic neighbor embedding (tSNE) plot for all samples with the major cell type populations denoted. FBIs, type 1 fibroblasts; FBIIs, type 2 fibroblasts; EBs, epidermal and basal cells; IMs, immune cells; VCs, vascular cells; NCs, neural crest derived cells; MIs, miscellaneous cells. **(B)** tSNE map with cells colored by time. **(C)** The expression levels of marker genes among cell types are shown in a violin plot. **(D)** The percentage of each cell type in UW skin and in WO skin at days 4, 8 and 14 (Wo4, Wo8, and Wo14). **(E,F)** Heatmap showing the major incoming or outgoing signals with the relative strength of each cell type in UW and WO skin. **(G,H)** River plot displaying the outgoing communication patterns of each cell type as well as the major signaling pathways in UW or WO skin. The thickness of the flow indicates the contribution of the cell type or signaling pathway to the corresponding pattern.

To build a high-resolution cell atlas of the wound healing process, we merged scRNA-seq libraries covering homeostasis and different repair stages of mouse back skin, including unwounded (UW) skin and wounded (WO) skin at 4 days (Wo4, GSE142471) 12, 8 days (Wo8, GSE108677) and 14 days (Wo14, GSE108677) 13. To obtain an unbiased and robust cell atlas, we used the *Seurat* package to perform batch correction and unsupervised clustering on 27827 cells (Figure 1A-B). Overall, 7 main cell types were determined based on the expression patterns of well-known marker genes (Figure 1A, C; Figure S1A-B; Table S1). Fibroblasts comprised the largest percentage of cells and were categorized into two types: type 1 fibroblasts (FBIs), which expressed genes involved in fibrosis and extracellular matrix (ECM) accumulation and were marked by high *Mfap4* and *Col1a1* expression, and type 2 fibroblasts (FBIIs), which expressed higher levels of immune factors, such as *Il6* and *Ccl2*, than FBIs. As previously established, the percentage of cells of each type, including *Krt14^+^* epidermal and basal cells (EBs), *Cdh5^+^* vascular cells (VCs), *Cd74^+^* immune cells (IMs), *Gpm6b^+^* neural crest derived cells (NCs), and *Pvalb^+^* miscellaneous (MIs), varied by stage (Figure 1D).

To verify the reliability of the integrated library, we applied *CellChat* to overview the major signaling inputs and outputs for cells in UW or WO skin 14. Macrophage migration inhibitory factor (MIF) signaling expression is reported to be implicated in cutaneous homeostasis and wound healing and plays a role in the regulation of immune activity 15. As described previously, EBs and NCs are important sources of MIF, which is mainly received by IMs in intact skin 16, 17 (Figure 1E). In WO skin, the expression of MIF in IMs is upregulated as the relative strength of outgoing signaling in IMs increases (Figure 1F), which is consistent with previous report 17. Complement activation, as a part of innate immune system activity, is induced by various tissue injury and is required for wound repair 18. According to our analysis, COMPLEMENT signaling was mainly released by FBIIs and acted on immune cells, which showed high strength in WO skin instead of UW skin (Figure 1E, F). We then employed pattern recognition in CellChat to reveal the main secretory signaling events in different cell types of skin. In UW skin, the majority of outgoing FBI signals were characterized by pattern #3 with high GALECTIN, PROS, IGF, ncWNT, and TWEAK levels (Figure 1G). While in WO skin, FBIs and FBIIs coordinated to send numerous signals for pathways such as the ANGPTL, MK, and CXCL pathways (Figure 1H). Interestingly, outgoing signaling by NCs exhibited the same pattern as that of IMs, which are well-established communication “hubs” in tissue repair, with largely overlapping signaling in both UW and WO skin (Figure 1G, H). *CellChat* analysis of these cell types also revealed that the interactions of NCs with FBIs, FBIIs and EBs were enhanced after injury, with increased interaction numbers or interaction strength (Figure S1C-D). Thus, our integrated scRNA-seq library over time provides a good in silico model that recaptures the heterogeneous cellular components of skin and the dynamic interactions of NCs with other cell types.

### DSCs are activated in injured skin

**Figure 2:**
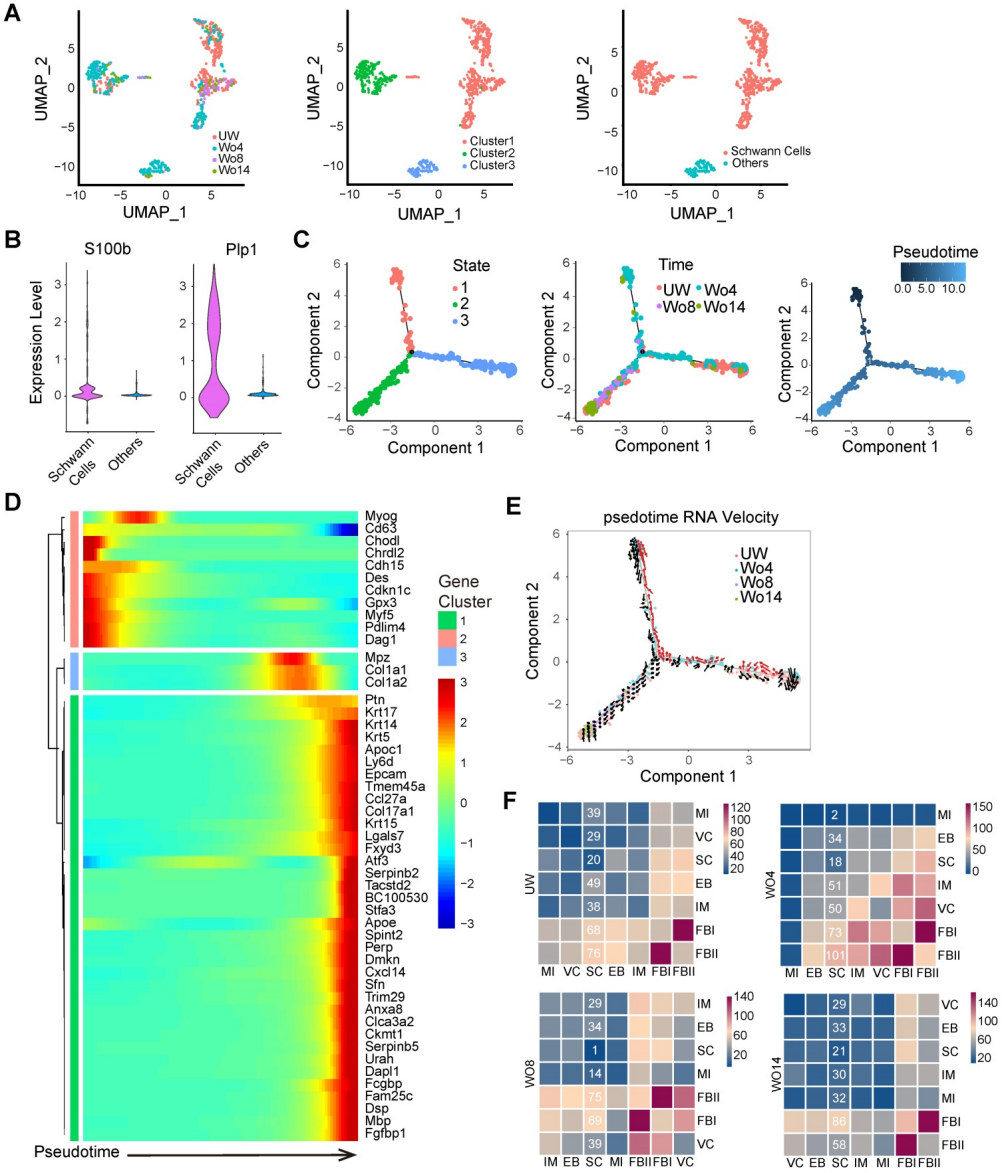
** Identification of SCs in UW and WO skin. (A)** UMAP analysis of NC transcriptomes with cells colored by time (left), Seurat cluster (middle) or cell type (right) identity. **(B)** Violin plots showing the expression levels of *S100b* and *Plp1* in the subpopulations of NCs. **(C)** Pseudotime analyses trace putative SC differentiation trajectories with cells colored by State (left), time (middle) or pseudotime (right) identity. **(D)** Heatmap showing the dynamically changing genes of all the cells used for the *Monocle* analysis arranged based on their pseudotime values. **(E)** State identities and RNA velocity vectors projected onto pseudotime trajectories of SCs. Velocity vectors are displayed as arrows, with the predicted dedifferentiation paths highlighted in red. **(F)** Heatmap of the total number of putative interactions between cell types in UW skin and WO skin at Wo4, Wo8 and Wo14.

Although some previous research has investigated the importance of neural cells in tissue regeneration, the mechanism of how neural cells respond to skin injury over time is not fully understood 2, 10, 11. To profile the subpopulation of NCs, we selected the NC subset from integrated datasets for uniform manifold approximation and projection (UMAP) analysis with unsupervised clustering in Seurat (Figure 2A). SCs, which were distinguished from other cells by their *S100b*^high^ and *Plp1*^high^ signatures, were a dominant subpopulation of NCs (Figure 2A-B). Pseudotime analyses of the SCs revealed distinct cell states across the pseudotime trajectories that indicated an active SC response to skin injury (Figure 2C). Intriguingly, the predicted SC precursors (State 1), which occupied the early stage of the pseudotime trajectory, were found mainly in the Wo4 population (Figure 2C). To further confirm the SC trajectory, we profiled significantly differentially expressed genes (DEGs) across pseudotime trajectories with *Monocle* packages (Figure 2D). Pseudotime trajectory recaptured the differentiation process of SCs, which were *Gpx3*^high^ at the beginning and expressed high levels of the differentiation marker *Mbp* at the end (Figure 2D). RNA velocity tracing suggested that State 1 SCs were derived from putatively mature SCs in UW skin (State 3), which indicated the dSCs in the early stage of wound healing (Figure 2E). According to the RNA velocity, SCs in State 2 that were enriched at Wo14 and occupied the differentiation stage of the pseudotime trajectory, may represent re-differentiated cells from State 1 (Figure 2E). Consistently, Wo4 SCs were marked by high *Gpx3* expression, while Wo14 SCs showed high *Mbp* expression (Figure S2A). Depending on the cell cycle score, SCs entered an active proliferative phase at approximately Wo8, with a low percentage of cells in the G1 phase and high *Mki67* and *Top2a* expression (Figure S2B- C). To infer communication between SCs and other cells at high resolution, we ran *CellPhoneDB* to evaluate the meaningful biological interactions at each time point. Intriguingly, the interactions of SCs with other cell types were increased in the Wo4 cell population, with most of them were related to FBs (Figure 2F; Figure S2D).

### DSCs defect in diabetic wounds

**Figure 3:**
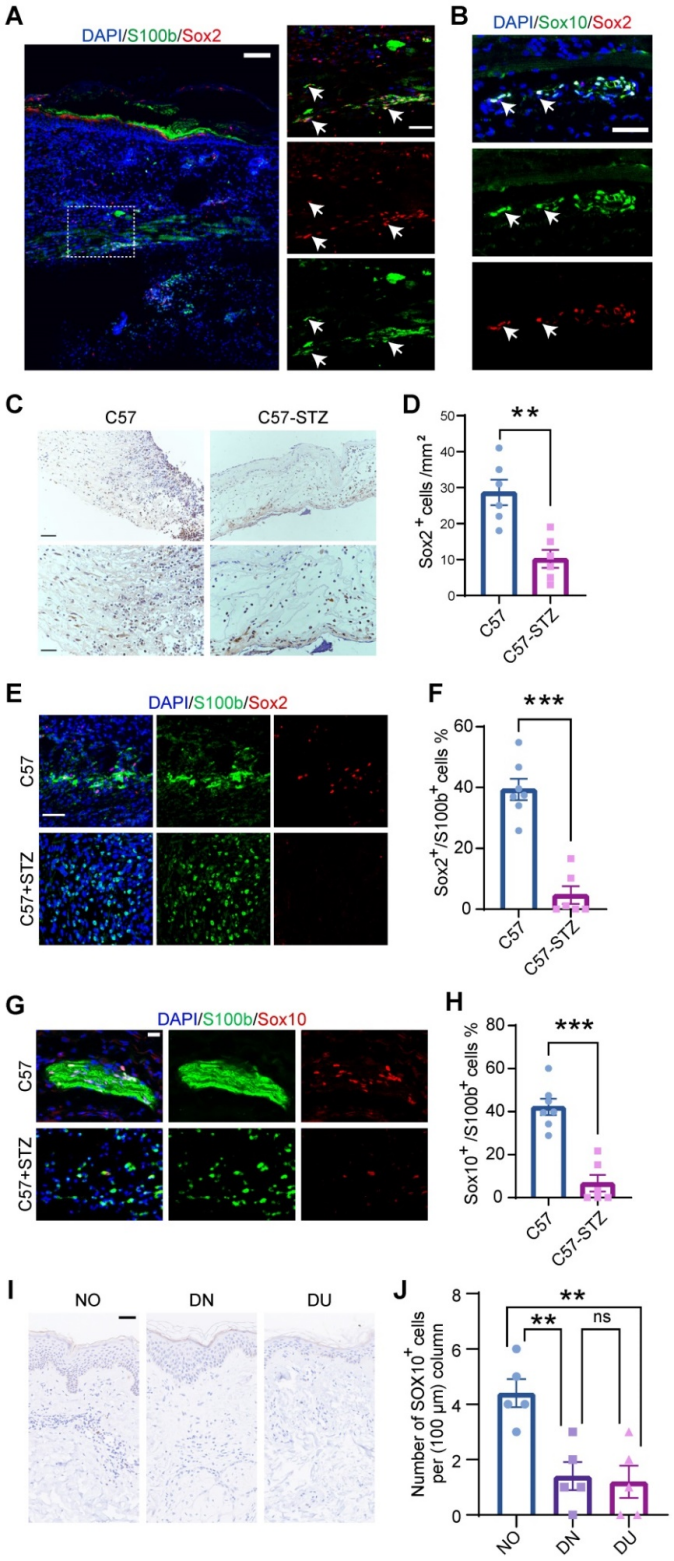
** Characterization of SCs in normal and diabetic wounds. (A)** Immunostaining of SCs labeled with S100b at D7. The higher-magnification image highlights the presence of Sox2^+^S100b^+^ dedifferentiated SCs (white arrowheads). Scale bars: 100 mm (left), 50 mm (right). **(B)** Immunostaining of Sox2 and Sox10 in regenerating skin at D7. Scale bars: 50 mm. **(C)** Representative images from immunohistochemical analysis of wounds from C57 control mice (C57) and mice with STZ-induced diabetes (C57-STZ) with Sox2 labeling at Wo7. Scale bar, 200 µm (up), 100 µm (down). **(D)** Quantification of Sox2^+^ cells in C57 or C57-STZ. **(E)** Immunostaining of Sox2 and S100b in skin sections from C57 and C57-STZ. Scale bar, 50 µm. **(F)** Quantification of Sox2^+^ cells among S100b^+^ cells in C57 or C57-STZ. **(G)** Immunostaining of Sox10 and S100b in wound slices from C57 and C57-STZ. Scale bar, 20 µm. (H) Quantification of Sox10^+^ S100b^+^ cells in C57 or C57-STZ. **(I)** Representative images of SOX10 immunostaining of diabetic skin in unwounded (diabetic non-wounded, DN) and wounded (diabetic ulcer, DU) regions and normal skin (NO). Scale bar, 50 µm. **(J)** Quantification of Sox10^+^ cells in unwounded and wounded regions of diabetic patients. The data are presented as the mean ± SEM, unpaired t test, *P < 0.05, **P < 0.01, ***P < 0.001.

To confirm SC dedifferentiation in WO skin, we took advantage of a splinted wound-healing model that is similar to skin regeneration in humans 19. Immunostaining showed that SCs expressing Sox2, a signature gene of dSCs that are characterized as SC precursors 10, existed in wounds at day 7 (D7) and were localized in the lower dermis (Figure 3A). Besides, Sox2^+^ dSCs were also marked by Sox10, a key signature of multipotent neural crest stem cells (Figure 3B). Peripheral neuropathy and chronic wounds are common complications in diabetic patients, but effective treatment is lacking 20. The relationship between SCs and abnormal wound healing in diabetic patients remains unclear. Since dedifferentiation is a vital program of SCs in wound healing, we observed Sox2^+^ cells in the wound area in C57 control mice (C57) and mice with streptozotocin (STZ)-induced diabetes (C57-STZ). There were fewer dSCs recognized by Sox2 expression in the C57-STZ wounds than in the C57 wounds (Figure 3C-D). Moreover, the morphology of the SCs in the diabetic mice was also obviously changed, with a lack of spreading and soma elongation, and the Sox2^+^ SCs were decreased (Figure 3E-F). The immunostaining of Sox10 in C57 and C57-STZ also reflected the reduced number of dSCs in diabetic wound (Figure 3G-H). To examine whether this phenotype is conserved in different species, we also analyzed single-cell sequencing data from diabetic patients (raw reads from published reports) 21 (Figure S3A-D). Although they were identifiable in diabetic skin wounds, the SCs displayed unconspicuous change in expression level of precursor genes, which indicated dysfunction of dedifferentiation (Figure S3E). Consistently, there were few dSCs labeled by SOX10 in diabetic skin, whether intact (diabetic non-wounded, DN) or wounded state (diabetic ulcer, DU), and normal skin (NO) (Figure 3I-J).

### Increased Wnt levels are required for SC dedifferentiation

**Figure 4:**
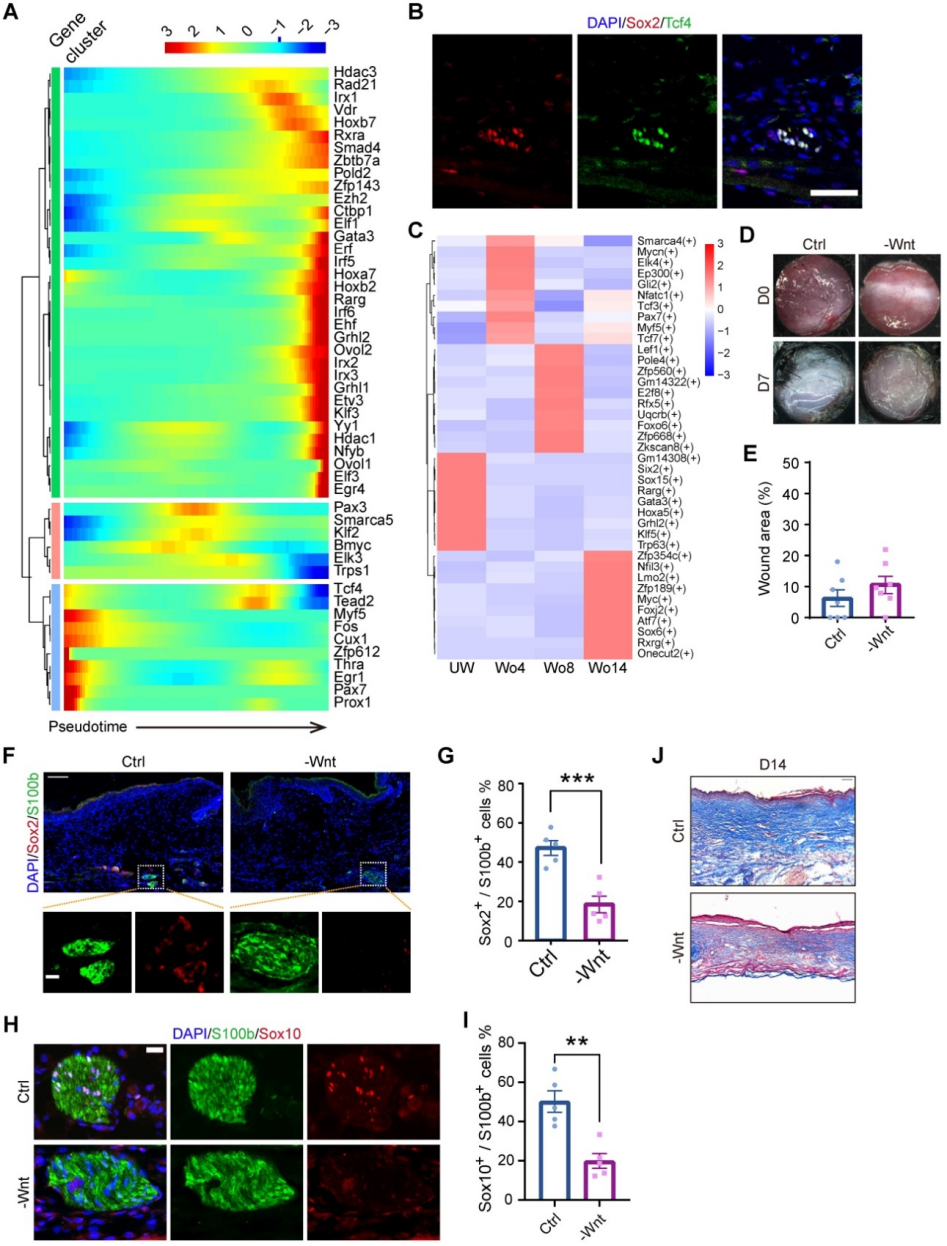
** Inhibition of Wnt signaling at an early stage impacts wound healing and decreases SC dedifferentiation. (A)** Heatmap showing the transcription factors with significantly changed expression among all the cells used for the *Monocle* analysis arranged by pseudotime values. **(B)** Immunostaining of Sox2 and Tcf4 in D7 wounds. Scale bar, 50 µm. **(C)** Heatmap showing the activity of the time-specific representative regulon at each time point. **(D)** Representative gross photographs of wounds treated with saline (Ctrl) or XAV939 (-Wnt) at D0 and D7. The inner diameter of the collar is 6 mm. **(E)** Quantification of individual wound areas at Wo7 in Ctrl and -Wnt animals relative to their initial sizes at D0. **(F)** Immunostaining of S100b and Sox2 in the skin of Ctrl and -Wnt wounds at D7. Scale bar, 100 µm. **(G)** Quantification of Sox2^+^ cells among S100b^+^ cells in Ctrl or -Wnt wounds. **(H)** Immunostaining of S100b and Sox10 in the skin of Ctrl and -Wnt wounds at D7. Scale bar, 20 µm. **(I)** Quantification of Sox10^+^ S100b^+^ cells in Ctrl or -Wnt wounds. **(J)** Representative images of skin sections with Masson staining from the denoted wounds at D14. Scale bar, 100 µm. The images of skin are representative of three independent experiments. The data are presented as the mean ± SEM, Unpaired t test, *P < 0.05, **P < 0.01, ***P < 0.001.

To further identify the mechanism of SC dedifferentiation, we assessed the expression levels of transcription factors (TFs) throughout the pseudotime trajectory. This analysis identified three gene clusters, with *Myf5* and *Tcf4* being significantly enriched in the precursor-state of SCs, s referred to dSCs (Figure 4A). Since the initial point of the pseudotime trajectory was mainly composed of cells at Wo4, we investigated the upregulated genes in Wo4 cells. Of note, Sox2+ dSCs were also marked by Tcf4 in D7 wounds (Figure 4B). Gene Ontology (GO) term analysis of the upregulated genes in Wo4 cells indicated that dedifferentiation of SCs was associated with upregulation of genes related to wound responses and immune regulation at an early stage (Figure S4A). To investigate the underlying mechanism of dedifferentiation, we performed transcriptome regulatory network analysis at each time point. Regulons implicated in the Wnt signaling pathway, such as the regulons targeted by *Myf5* and *Tcf3* 22, were activated to markedly high levels in Wo4 cells (Figure 4C). Tcf3 and Tcf4 are the key factors that collaborate with β-catenin to regulate wound repair of both the epidermis and hair follicles 23. Consistently, *Myf5*, which is a signature gene of neural crest-like precursors 24, was specifically upregulated in SCs at Wo4 when dedifferentiation occurred (Figure S4B). Activated Wnt signaling in dSCs was also confirmed by RNA sequencing (RNA-seq) analysis of SCs (raw RNAseq reads from published data 11) in the early state of wound healing (Figure S4C-D).

To determine whether Wnt signaling is necessary for SC dedifferentiation, WO mice were subcutaneously injected with the Wnt inhibitor XAV939 after lesion induction to repress Wnt in the dermal region of the wound bed. At Wo7, hematoxylin and eosin (H&E) staining showed that Wnt inhibition (-Wnt) mice displayed mild defects in wound closure with no significant change in wound size relative to that of control (Ctrl) mice (Figure 4D-E). However, there was a significant reduction in Sox2^+^ dSCs in the -Wnt mice (Figure 4F, G). Consistently, the proportion of Sox10^+^ SCs in -Wnt mice was lower than that in Ctrl mice (Figure 4H-I). Since collagen genes were upregulated in SCs at later stage (Figure 2D, Figure S2E), we wondered whether SCs also contribute to ECM protein deposition. According to Masson staining at Wo14, -Wnt mouse tissues contained less collagen than sham mouse tissues, possibly due to SC dysfunction (Figure 4J). Together, these findings suggest that upregulated Wnt signaling is needed for the dedifferentiation of SCs.

### DSCs that secrete TGF-β3 at an early state are required for wound healing

**Figure 5:**
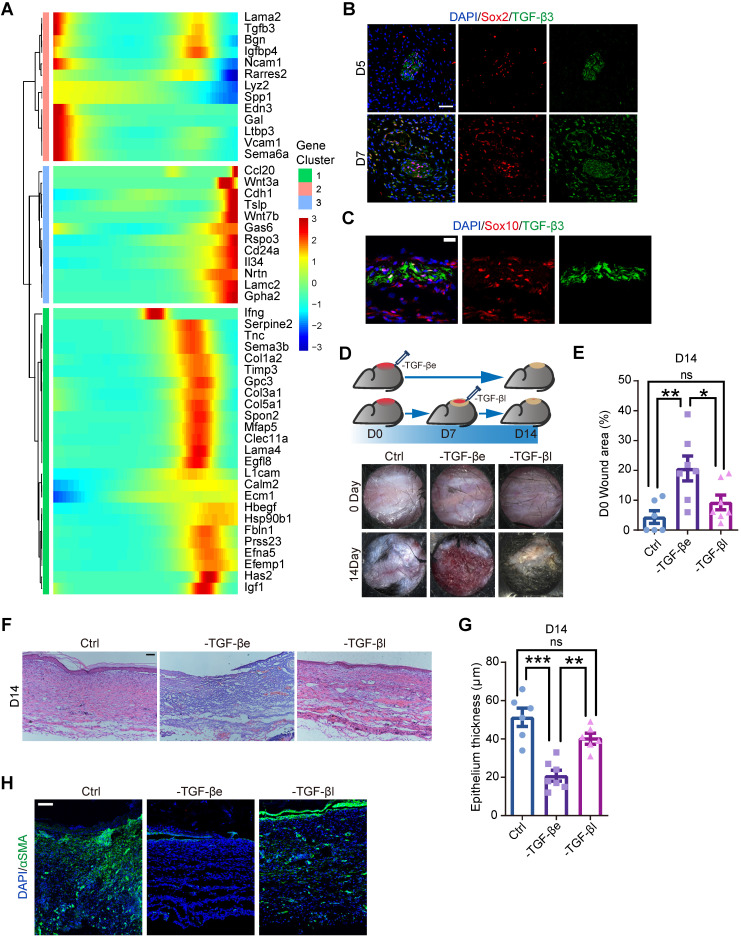
** TGF-β inhibition *in vivo* at an early stage causes improper wound healing. (A)** Heatmaps showing the pseudotime-dependent genes encoding ligands of all SCs across the pseudotime trajectory. **(B)** Immunostaining of TGF-β3 and Sox2 in the dermal region in D5 and D7 wounds. Scale bar, 50 µm.** (C)** Representative images of Sox10 and TGF-β3 immunostaining in the dermal region in D5 wounds. Scale bar, 20 µm.** (D)** Schematic (top lane) and gross images (bottom lane) of wounds treated with saline (Ctrl) or the TGF-β inhibitor SB431542 at D0 (-TGF-βe) and D7 (-TGF-βl). **(E)** Quantification of the relative wound size in the denoted wound types at D14. One-way ANOVA. **(F)** Hematoxylin and eosin (H&E) staining histology of Ctrl, -TGF-βe (-TGF-β early) and - TGF-βl (-TGF-β late) D7 wounds. Scale bar, 100 µm. **(G)** Quantification of the epidermal thickness of Ctrl, -TGF-βe and -TGF-βl wounds. One-way ANOVA. **(H)** Representative images of sections from Ctrl, -TGF-βe and -TGF-βl wounds immunostained for aSMA. The images are representative of three independent experiments. Scale bar, 100 µm. The data are presented as the mean ± SEM, *P <0.05, **P < 0.01, ***P < 0.001.

Previous research has revealed that SCs support tissue regeneration by secreting growth factors 10, 11. Consistently, we found that several genes encoding growth factors were expressed in SCs, including *Pdgfa*, as previously reported 10 (Figure S2D, S5). To explore the molecular mechanism by which SCs regulate wound healing, we searched for ligand-encoding genes that exhibited pseudotime dynamics. Since dSCs were activated at an early stage, we sought secreting factors that were highly activated in State 1, corresponding to Wo4 (Figure 5A). According to previous suggestions that SCs enhance TGF-β signaling during wound healing 11, we considered TGF-β3, which is encoded by *Tgfb3*, is most likely to be the key factor. Immunolabeling of wound sections from mice displayed the presence of TGF-β3^+^ dSCs as early as D5 (Figure 5B-C). To examine whether TGF-β signaling activation at an early stage is critical for appropriate wound healing, we performed full-thickness punch wounds on the back skin of mice and treated them with TGF-β inhibitor at D0 (-TGF-β early, -TGF-βe) or D7 (-TGF-β late, -TGF-βl) (Figure 5D). Relative to the -TGF-βl mice, failure of wound closure and epithelialization were more severe in the -TGF-βe mice (Figure 5D-G). Moreover, among the three treatment groups, the -TGF-βe mouse group showed the least aSMA expression (Figure 5H). Thus, we conclude that dSCs contribute to increased TGF-β3 levels in the deep dermal layer at an early stage, which is critical for appropriate repair.

### DSCs promote cell migration by secreting TGF-β3

Keratinocytes and fibroblasts are major cell types in skin regeneration and express receptors corresponding to SCs (Figure S1D, S2D). To test whether dSCs promote wound healing via secreting factors, we applied a cell culture system with keratinocyte and fibroblast cell lines and medium from different SC cell lines. Since immortalized cultured SCs express myelin genes at low levels comparable to those observed *in vivo*, we considered the SC cell lines to be immature SCs, which are dSC in wound 25. In detail, mediums from RSC96 and S16 cells were harvested and added to NIH-3T3 (3T3) and HaCaT cells. After 1 day of treatment, there was little change in the proliferation of 3T3 and HaCaT cells, as determined by the percentages of EdU^+^ cells (Figure S6A, B).

**Figure 6:**
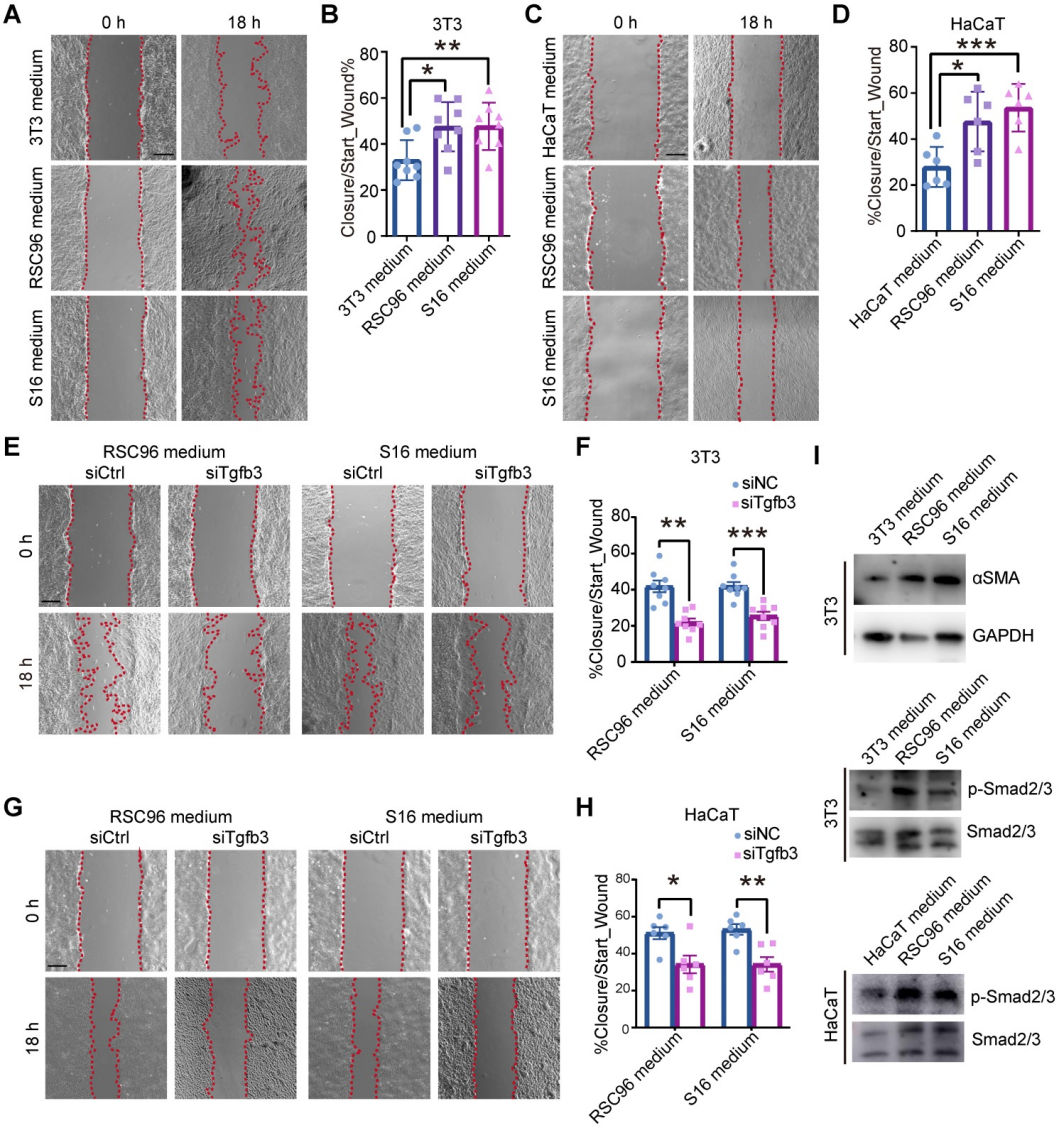
** SCs promote the migration of fibroblasts and keratinocytes. (A)** *In vitro* wound-healing assays of 3T3 cells cultured in mediums from 3T3, RSC96 or S16 cells. **(B)** Migration rates were evaluated according to the closure area at 18 h after lesion induction. **(C)** *In vitro* wound-healing assays of HaCaT cells cultured in mediums from HaCaT, RSC96 or S16 cells. **(D)** Quantification of wound closure at 18 h after scratching. **(E)** *In vitro* wound-healing assays of 3T3 cells cultured in medium from RSC96 or S16 cells after control or *Tgfb3* RNAi (siCtrl or siTgfb3) treatment. **(F)** Quantification of wound closure of 3T3 cells under different conditions. **(G)** *In vitro* wound-healing assays of HaCaT cells cultured under the indicated conditions. **(H)** Quantification of wound closure in HaCaT cells under the indicated conditions. **(I)** Immunoblot analysis of the expression of aSMA, phosphorylated Smad2/3 (p-Smad2/3) and Smad2/3 in 3T3 or HaCaT cells after incubation with mediums from RSC96 or S16 cells for 24 h. The images presented are representative of three independent experiments. Scale bars: a, c, e, g, 200 mm. Unpaired t test. The data are presented as the mean ± SEM, *P<0.05, **P < 0.01, ***P < 0.001.

Previous research has suggested that the migration of fibroblasts from the lower dermis and keratinocytes into the wound bed is a critical event in the early phase of repair 26, 27. At the wound edge, the recruitment of epithelial cells is initiated within 12 h after injury 27, 28. GO enrichment analysis of the highly expressed genes of fibroblasts and EBs at Wo4 also supported the suggestion that fibroblasts and keratinocytes display active migration in the early stage (Table S2). To assay the effect of dSCs on migration, we performed an *in vitro* wound-healing assay with 3T3 and HaCaT cells incubated in RSC96 and S16 medium. The increased covered area indicated that dSCs enhanced the migration of dermal and epidermal cells (Figure 6A-D).

We then investigated whether TGF-β3 is the key factor that determines the effects of dSCs on keratinocyte and fibroblast motility. First, we identified an appropriate interfering RNA (si2) that could inhibit TGF-β3 expression in both SC cell lines (Figure S6C). Then, the medium from the TGF-β3-deficient cell culture was used to incubate 3T3 and HaCaT cells during the migration assay. The results indicated that TGF-β3, inhibition repressed the effects of SCs on keratinocytes and fibroblasts (Figure 6E-H)*.* Compared with cells exposed to medium from SCs after RNAi for TGF-β1 or TGF-β2, which showed low expression level in Wo4 SCs (Figure S5), the migration was decreased in cells exposed to the medium from TGF-β3-deficient SCs (Figure S6D-G). Consistently, the expression level of aSMA, which is implicated as a downstream gene of TGF-β3, was increased, and the phosphorylation levels of Smad2/3 were upregulated in cells incubated in SC culture medium (Figure 6I). These findings suggest that TGF-β3 mediates the effect of dSCs on the migration of surrounding cells in WO skin.

### TGF-β3 in the wound bed is a potential strategy to enhance regeneration in diabetic wounds

**Figure 7:**
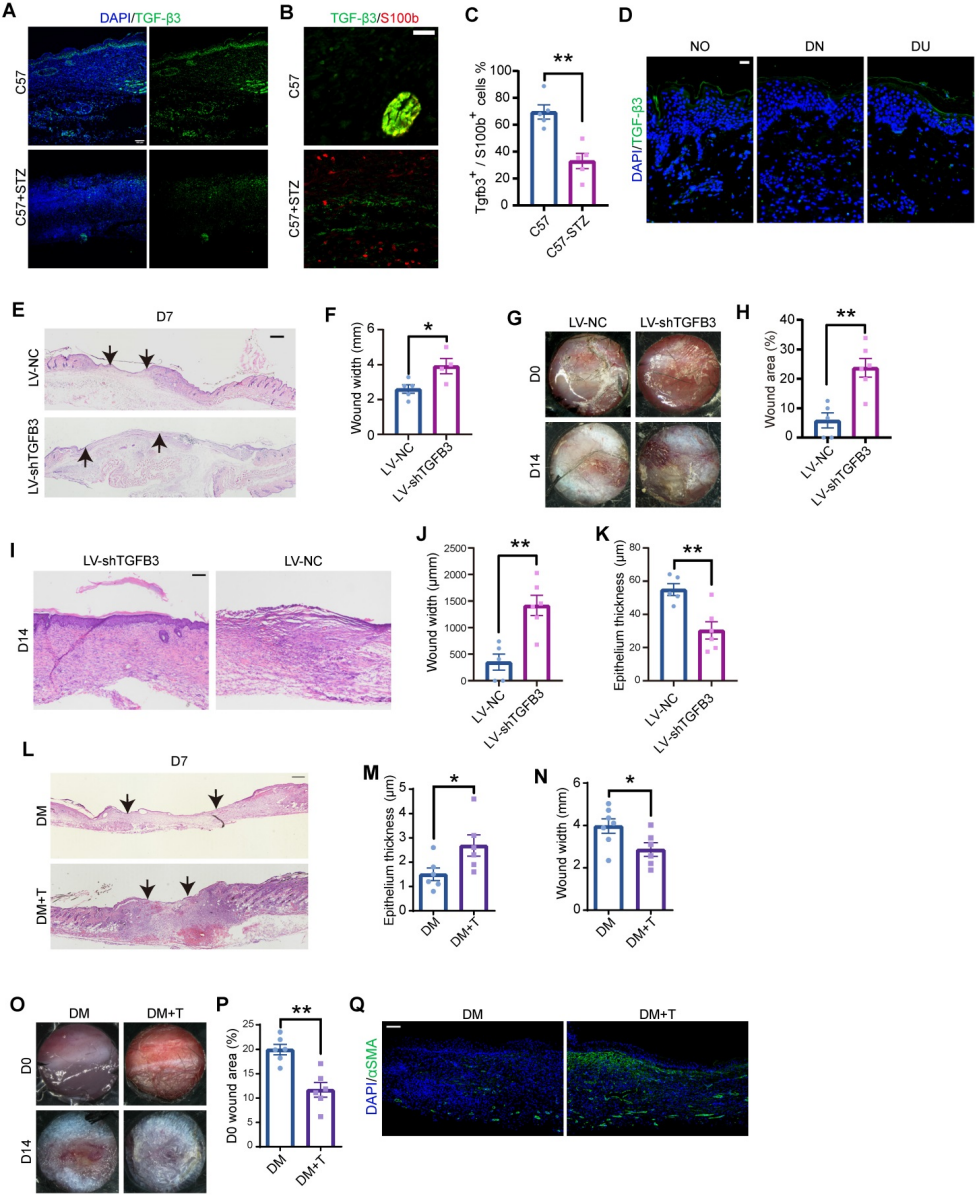
** Analysis of the role of TGF-β3 in diabetic wounds. (A)** Immunostaining of TGF-β3 in sections from C57 and C57-STZ wounds at D7. Scale bar, 100 mm. **(B)** Representative images of S100b and TGF-β3 immunostaining in the dermal region in D7 wounds. Scale bar, 50 µm.** (C)** Quantification of TGF-β3^+^ S100b^+^ cells in C57 or C57-STZ.** (D)** Immunostaining of TGF-β3 in intact skin from non-diabetic human (NO) and unwounded or wounded skin from diabetic patients (DN and DU). Scale bar, 100 mm. **(E)** Representative H&E staining of wounds from mice injected with control (LV-NC) or Tgfb3 RNAi (LV-shTgfb3) virus at D7. Scale bar, 1 mm. **(F)** Quantification of the wound width of LV-NC and LV-shTgfb3 wounds at D7. **(G)** Representative gross images of wounds from LV-NC or LV-shTgfb3 mice D0 and D14. **(H)** Quantification of individual wound areas at D14 in LV-NC and LV-shTgfb3 mice relative to their initial sizes at D0. **(I)** H&E staining of LV-NC and LV-shTgfb3 wounds at D14. Scale bar, 1 mm. **(J,K)** Quantification of the wound width (J) and epithelial thickness (K) of LV-NC and LV-shTgfb3 wounds at D14. **(L)** H&E staining of diabetic wounds with or without TGF-β3 injection (DM+T and DM wounds) at D7. Scale bar, 1 mm. **(M,N)** Quantification of the wound width (M) and epithelial thickness (N) of DM and DM+T wounds at D7. **(O)** Representative images of DM and DM+T wounds at D14. **(P)** Quantification of wound size for DM+T wounds versus DM wounds. **(Q)** Representative images of aSMA staining on sections from DM and DM+T wounds at D14. Scale bar, 100 µm. Unpaired t test. *P < 0.05, **P < 0.01, ***P < 0.001.

Consistently, the TGF-β3 expression level was lower in C57-STZ cells than in C57 cells in the deep dermal layer, where SCs are located (Figure 7A-C). Immunostaining of diabetic ulcers also revealed TGF-β3 deficiency in the dermal layer (Figure 7D). We then wondered whether dSC dysfunction contributes to chronic wounds in diabetic patients. To specifically inhibit the SC derived TGF-β3, we applied GFAP-promoter driven *Tgfb3* RNAi by virus injection (Figure S7A-B). H&E staining and gross observation indicated that repression of SC derived TGF-β3 led to a delayed wound repair with a greater wound width and lager wound area remained (Figure 7E-J). Moreover, TGF-β3 inhibition in SCs caused defects in re-epithelialization in later stage (Figure 7I, K).

Since dSC dysfunction and TGF-β3 deficiency are prominent in diabetic wounds, we questioned whether TGF-β3 addition, as a strategy to enhance the function of dSCs, can improve chronic wound healing. Diabetic mice were injected with TGF-β3 into the dermal layer and harvested at Wo7 or Wo14. H&E staining supported that wounds in diabetic mice treated with TGF-β3 by intradermal injection displayed greater wound closure and epithelialization than wounds in diabetic mice without TGF-β3 treatment (Figure 7L-N). A gross examination revealed that after intradermal TGF-β3 injection, the diabetic wounds were smaller with intradermal TGF-β3 injection than without TGF-β3 treatment at D14 (Figure 7O-P). Moreover, TGF-β3-treated wounds expressed higher levels of aSMA than wounds in diabetic model mice (Figure 7Q). Taken together, these data suggest that dSC dysfunction is related to chronic wounds in diabetic skin and that enhancing the function of dSCs is a promising therapeutic strategy.

## Discussion

Although many wound healing therapies have been developed, they have generated only moderate effects, which makes it difficult to solve complicated problems in numerous clinical cases. One reason for these unsatisfactory outcomes is the limited knowledge about the detailed roles of each cellular and molecular mechanism. The existence of a link between the nervous system and tissue regeneration has long been acknowledged 29. SCs are important component of the peripheral nervous system in skin and have been studied from a developmental perspective 30, 31. However, because they are a relatively small population, SCs have received less concerned in previous studies on skin regeneration. Single-cell techniques that investigate the transcriptome or genome of each cell in a specific region can be used to identify the cellular composition of a specific tissue and decipher the underlying mechanisms of different cells simultaneously. To gain better insight into the wound healing process, we generated a single-cell transcriptome atlas across different stages by anchoring diverse datasets together (Figure 1A-B). Our analysis indicates that SCs constitute the major neural cell type involved in skin repair (Figure 2A). Moreover, we found that the dedifferentiation of SCs occurs at an early stage with probably subsequent redifferentiation in silico (Figure 2C, E). Intriguingly, we also found that SCs express distinct secretomes in different stages (Figure 5A), which suggests that SCs may be key regulators that orchestrate multiple activities.

Cell plasticity that enables SCs to undergo dedifferentiation following injury is considered to be the key factor promoting repair 32. However, the underlying mechanism that regulates the dedifferentiation of SCs in response to injury remains largely unclear. Whether SCs display distinct repair processes in different tissues is also unknown. The Raf/MEK/ERK signaling pathway is recognized as an intracellular mediator in the response of SCs to nerve injury, leading to activation of Ras kinase activation 33. Although most activated dSCs after injury can be identified by c-Jun expression, overexpression of c-Jun in SCs is insufficient to initiate a repair program 34. We identified Wnt activation in dSCs by analyzing the transcriptome regulatory network at the single-cell level and the gene expression profile of activated dSCs (Figure 4A, C; Figure S4B- D). *In vivo* Wnt inhibition blocked the dedifferentiation of SCs (Figure 4F-I), supporting the notion that Wnt is indispensable for dedifferentiation. This finding is consistent with previous suggestions that Wnt signaling is critical in wound healing 35. Previous research has demonstrated the function of Wnt signaling in the development of SCs. Our results further extend our understanding of the function of Wnt signaling in dSCs.

Increasing evidence indicates that SCs regulate nonneural tissue stem cell populations via secreting paracrine factors 8. Nonmyelinating SCs are key components of the bone marrow niche and maintain hematopoietic stem cell hibernation by regulating latent TGF-β 36. A previous study has suggested that dSCs in injured tissue promote mesenchymal cell proliferation and blastema expansion by secreting oncostatin M and PDGF-AA 10. To assess the effects of dSCs on keratinocytes and fibroblasts, we took advantage of the RSC and S16 cell lines, which are recognized as immature SC lines. According to our experiments, dSCs promote the migration of keratinocytes and fibroblasts but have little effect on the proliferation of these cells (Figure 6A-D, Figure S6A, B). We suggest that SCs need other factors at the injury site to drive proliferation, while immature SCs regulate the migration of surrounding cells. Injury-activated keratinocytes form the migrating epithelial tongue and migrate into the wound site for re-epithelization 37. Compared with adult fibroblasts, fetal fibroblasts migrate faster, which contributes to their highly efficient regeneration 38. Thus, we propose that the function of SCs in tissue regeneration involves more than enhancement of the proliferation of stromal cells.

The TGF-β pathway is one of the pivotal signaling pathways in tissue regeneration, with diverse effects depending on the specific environment and the molecules involved 39. TGF-β3 is an important ligand that guides the migration of various cells to coordinate well-organized motility in wound healing 40, and participates in the inflammatory regulation of scar production 41, 42. Previous reports have shown that dSCs in the wound site promote myofibroblast formation via paracrine modulation of TGF-β signaling 10. According to our analysis, TGF-β3 is the key TGF-β ligand expressed in dSCs at an early stage. The inhibition of TGF-β3, but not TGF-β1 or TGF-β2, inhibition *in vitro* blocked the effect of dSCs on cell motility (Figure 6E-H, S6D-G). In addition, the results of our *in vivo* experiments are in line with a previous proposal suggestion that TGF-β signaling activation is pivotal in the early wound-healing stage 43. Thus, we propose that dSCs are the critical sources of TGF-β3 at an early stage and that they model TGF-β signaling in the microenvironment.

It has been well established that innervation and neurogenic factor deficiency are related to chronic wounds in diabetic patients. Schwannopathy, an integral factor in the pathogenesis of diabetic neuropathy, is also involved in chronic diabetic wounds. We found decreased numbers of dSCs with abnormal morphology in the chronic wounds of diabetic mice, and these abnormalities were accompanied by TGF-β3 deficiency in the dermal layer (Figure 7A-C). Moreover, TGF-β3 defects during wound healing were conserved in diabetic patients, as corroborated by immunostaining (Figure 7D). Consistently, conditional downregulation of SC-derived TGF-β3 cause poor regeneration in mice (Figure 7E-K). In contrast, increasing the levels of local TGF-β3 in diabetic wounds improved wound repair in diabetic mice (Figure 7L-Q). We therefore propose that these regeneration deficits in diabetic patients are due, at least in part, to dSC dysfunction.

**Figure 8:**
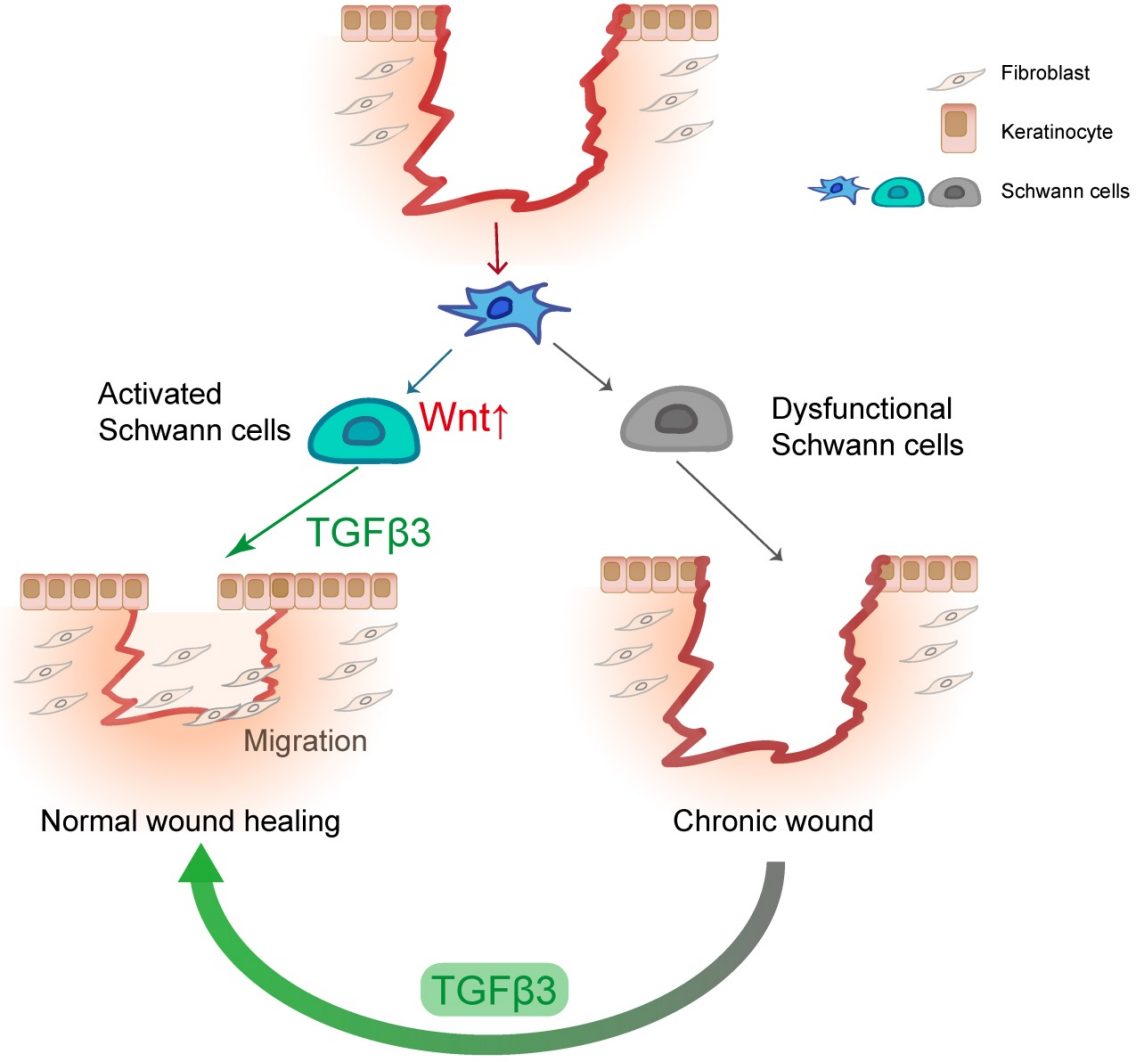
** SCs facilitate wound healing by releasing TGF-β3.** In response to injury, dermal SCs around the wound region undergo dedifferentiation with Wnt activation, contributing to wound healing by promoting migration of fibroblast and keratinocyte. In diabetic skin, dysfunctional SCs are failed to secrete TGF-β3, which could be a therapeutic strategy for chronic wound.

This study advances our understanding of the role of SCs in wound healing (Figure 8). In line with previous findings, we identified that the dedifferentiation of SCs occurs in the early stage, which may be part of the rapid wound response of the injured site. Our experiments demonstrate that Wnt signaling is necessary for the dedifferentiation of SCs. We found that dSCs promote the migration of keratinocytes and fibroblasts by secreting TGF-β3, which is specifically and highly expressed in dSCs. Moreover, we found that TGF-β3 is capable of improving diabetic wound healing. Considering our findings and those of previous studies, we suggest that the activity of dSCs is an essential part of wound healing and a potential target for improving improper skin repair.

## Materials & Methods

### Human skin samples

Diabetic wound sections were collected from diabetic ulcer samples. Diabetic skin samples were collected from surgery with informed patients following protocols and institutional ethics guidelines approved by the ethics committee of Shanghai Ninth People's Hospital.

### Animals

The mouse experiments were performed on 6- to 8-week-old wild-type C57BL/6J mice, which were bred under standard conditions. All mouse experiments followed protocols approved by Shanghai Ninth People's Hospital.

### Induced diabetes in mice

To induce pancreatic islet β-cell impairment and diabetes, male C57BL/6 mice (20-22 g) were injected with STZ (150 to 200 mg/kg) at least 1 week before wounding. Mice were recognized as diabetes when their blood glucose levels were greater than 16 mmol/l.

### Wounding experiments

We used splinted wound model in all our mouse experiments. After anesthetization with isoflurane and shaving to clean hair, mice were disinfected with scissors, and a single full-thickness excisional wound 6 mm in diameter was generated on the dorsal skin. A silicone loop was sutured tightly adhering to the skin around the wound site, as previously described 19. Both male and female mice were used in the experiments. For Wnt inhibition at an early stage, 150 ml of XAV939 (1 mg/mL) was injected locally into the wound site after lesion induction, and another injection at the same dose was given on D3. For TGF-β inhibition in wound healing, 150 ml of SB431542 (5 mg/mL) was injected locally on D0 (iTGF-βe) or D7 (iTGF-βl). For TGF-β3 treatment, 200 ng of TGF-β3 (1 mg/mL) or placebo was injected immediately following injury creation. After mouse sacrifice, skin from the wound and surrounding regions was collected (approximately 1.5 cm diameter) for subsequent analysis.

### Virus transfection

To specifically repress the gene expression in SC, we applied lentivirus with GFAP-promoter for conditional *Tgfb3* RNAi. Control or *Tgfb3* RNAi (150 ml, ~1×10^7^ TU/mL) was injected into the the wound site 3 days before lesion, and another injection at the same dose was given on D0.

### Cell culture

NIH3T3 cells, HaCaT cells, and the SC lines RSC96 and S16 were maintained at 37 °C with 5% CO_2_ in DMEM medium supplemented with 10% FBS (Gibco) and 1% antibiotic-antimycotic (Gibco). To avoid mycoplasma contamination, the cells were examined by cytoplasmic DAPI staining at every passage. NIH3T3 cells, HaCaT cells, and RSC96 or S16 SCs mediums for incubation were collected from cells at 60-70% confluence cultured in DMEM without serum for 24 h.

### *In vitro* wound-healing assay

For wound-healing assays, HaCaT or NIH3T3 cells were seeded in 6-well culture plates with complete medium to form cell monolayers at almost 100% confluence. The medium of the cells was replaced by DMEM without FBS for serum deprivation 16 h before wounding. A 200 μl pipette tip was used to scratch the HaCaT or NIH3T3 cell monolayers for lesion formation. After aspirating the medium and cell debris gently, the HaCaT or NIH3T3 cells were cultured in HaCaT, NIH3T3, RSC96 or S16 medium and photographed at 0 and 18 h. Wound closure was measured as follows: % wound closure = ((0 h wound area - 18 h wound area) × 100)/0 h wound area.

### *In vitro* proliferation assay

HaCaT or NIH3T3 cells were seeded in 12-well plates to approximately 30% confluence and cultured in 10% FBS and medium collected from HaCaT, NIH3T3, RSC96 or S16 cells in DMEM (1:1 mix). Then, 10 μM 5-ethynyl-2'-deoxyuridine (EdU) was added to the medium 2 h prior to analysis. EdU was detected using a BeyoClick™ EdU kit (Beyotime, C0071S).

### TGFB3 RNAi

Antisense oligonucleotides targeting *TGFB3* and a negative control duplex were purchased from RIBOBIO. To downregulate *TGFB3* specifically, cells were transfected with siRNA molecules or negative control via LipoRNAi™ Transfection Reagent (Beyotime, C0535) following the manufacturer's instructions for 24 h and then collected for subsequent Western blotting or cultured in DMEM for medium collection.

### Histological analysis

Skin tissue from humans or mice was fixed with 4% PFA overnight and dehydrated in 20% sucrose in PBS. Then, the tissues were embedded in Tissue-Tek O.C.T. Compound (Sakura Finetek, 4583) and cryosectioned (10-15 μm). H&E staining was performed with a Hematoxylin and Eosin Staining Kit (Beyotime, C0105) according to the manufacturer's instructions. For Masson trichrome staining, cutaneous sections were rehydrated through a series of 100% alcohol, 95% alcohol and 70% alcohol and then washed with ddH_2_O wash. Briefly, sections were stained with potassium bichromate solution (10 min), Ponceau-acid fuchsin solution (10 min) and aniline blue solution (10 min). After quick rinsing with ddH_2_O, the slides were differentiated with 1% acetic acid solution (2-5 min) and dehydrated through a series of 95% alcohol and 100% alcohol. After xylene treatment, the slides were mounted with resin.

### Immunostaining and immunoblotting

Skin sections were incubated in antigen retrieval solution (Beyotime, P0088) for 25 min at 95 °C and washed with PBS. After blocking in staining buffer (2% BSA+0.05% Triton in PBS) for 1 h, the slices were incubated with primary antibodies diluted in staining buffer overnight at 4 °C. Then, after 3-5 washes with PBS, the slices were incubated in staining buffer containing secondary antibodies for 1 h at room temperature. For immunofluorescence of cultured cells, cells adherent to slices were fixed with 4% PFA for 15 min at room temperature and permeabilized in 0.25% Triton X-100 in PBS for approximately 10 min. The staining process for cell immunofluorescence was similar to that for tissue sections except for staining buffer did not contain Triton X-100.

After washing quickly in PBS, the cells were harvested by scratching and lysed in RIPA buffer (50 mM pH 7.4 Tris-HCl, 150 mM NaCl, 1% NP-40, 0.5% sodium deoxycholate) containing Protease Inhibitor (Beyotime, P1005) for 20 min on ice. After denaturation in 5x SDS sample buffer for 10 min at 95 °C, the protein lysates were subjected to SDS-PAGE and transferred to 0.45 μm PVDF membranes. After incubation in 5% BSA in TBST, the PVDF membranes were incubated in dilution buffer (2% BSA in TBST) containing primary antibodies overnight at 4 °C and then incubated with HRP-conjugated secondary antibodies diluted in dilution buffer. The proteins were recognized with the following antibodies: rabbit anti-S100 (DAKO, Z0311), mouse anti-S100b (Atlas Antibodies AB, AMAB91038), goat anti-Sox-2 (Santa Cruz, sc17319), mouse anti-Sox10 (Abcam, ab216020), rabbit anti-Sox10 (Abcam, ab180862), rabbit anti-TGFb3 (Abcam, ab15537), rabbit anti-Smad2/3 (Affinity, AF6367), rabbit anti-phospho-Smad2/3 (Affinity, AF3367), mouse anti mCherry (Abcam, ab125096) and rabbit anti-aSMA (Abcam, ab5694).

### ScRNA-seq and RNA-seq data analyses

For mouse wound healing analysis, the BAM files of GSE142471 12 and GSE108677 13 were downloaded from NCBI and then converted to fastq files by *bamtofastq* (v1.3.2). The expression matrix of each original file generated with *CellRanger* (v4)* count* with the default parameters was integrated and normalized by *Seurat* (v3). In detail, we used *FindIntegrationAnchors* and *IntegrateData* in Seurat to prevent batch effects as followed code:

sce.anchors <- FindIntegrationAnchors(object.list = list(uw,uw1,uw2,wo1,wo2,wo3,wd8,wd14),dims = 1:20)

sce.combined <- IntegrateData(anchorset = sce.anchors, dims = 1:20)

DefaultAssay(sce.combined) <- "integrated"

To filter out low-quality cells before clustering, we kept cells showing >500 and <3000 RNA features and no more than 10% mitochondrial gene expression. The top 15 principal components (PCs) were used to assign cells to a UMAP space. Cell clusters were identified with the *FindClusters* function of *Seurat* (resolution = 0.6). Clusters that were proximal to each other in the UMAP space and shared similar transcriptional profiles were attributed to putative cell types, which were further identified by general markers. Cell cycle analyses were investigated by evaluating cell cycle-related genes with CellCycleScoring in *Seurat*. The differential trajectories of SCs were depicted by Monocle (v2). Then, pseudotime-dependent genes that significantly changed across pseudotime, were identified by *DifferentialGeneTest* in *Monocle*. To find pseudotime-dependent ligands and TFs, we searched ligand genes (from *Celltalker*) or TF genes (https://github.com/aertslab/pySCENIC/blob/2919ae49d124ddceb8a1c296b2d1d37c3e851325/resources/mm_mgi_tfs.txt) among the pseudotime-dependent genes. We used *velocyto* in Python to generate loom files and then projected velocity on the pseudotime trajectory with *velocity.R 44*. The velocity was calculated in the UMAP reduced space and then projected onto the *Monocle* pseudotime trajectory. To analyze the differences between the UW and WO groups, we employed *CellChat* to delineate intercellular communication networks and major signals from the scRNA-seq data. To investigate how multiple cell types and signals coordinate by communication, we ran the pattern recognition module in CellChat in either the UW or WO group. Communication between SCs and other cell types was estimated by *CellPhoneDB* software with the expression matrix and metadata from *Seurat*. The gene regulatory networks of cells were investigated by *pyscenic* analysis with the recommended pipeline in Python 45. For GO enrichment analysis of specific cell populations, we used the enrichGO function in *ClusterProfiler* to interpret the significantly upregulated genes. For diabetic wound analysis, the scRNA count matrix of GSE154557 21 was processed to cluster and profile gene expression in *Seurat*. The raw reads of PRJEB22372 from the European Nucleotide Archive were aligned to the hg19 human genome reference with *HISAT2*. After assembly with *StringTie*, the DEGs were identified by *Ballgown* 46.

### Statistics

Statistical analysis and plotting were performed in the R package. Two-tailed unpaired t tests were used for comparisons between two groups of data with a normal distribution, and ANOVA was used for multiple comparisons. All statistical details of the experiments are denoted in the figure legends. The data are presented as the mean ± SEM. *P < 0.05, **P < 0.01, ***P < 0.001.

## Supplementary Material

Supplementary figures and tables.

## Abbreviations

SCs: Schwann cells
scRNA-seq: single-cell RNA sequencing
NCs: neural crest derived cells
dSC: dedifferentiated SC
EdU: 5-ethynyl-2'-deoxyuridine
UW: unwouded
WO: wounded
Wo4: wounded 4 days
FBI: type 1 fibroblasts
ECM: extracellular matrix protein
FBII: type 2 fibroblasts
EBs: epidermal and basal cells
VCs: vascular cells
IMs: immune cells
Mis: miscellaneous
MIF: Macrophage migration inhibitory factor
UMAP: uniform manifold approximation and projection
D7: day 7
STZ: streptozotocin
C57: C57 control
C57-STZ: streptozotocin (STZ)-induced diabetic mice
DEGs: differentially expressed genes
GO: Gene Ontology
TFs: transcription factors
RNA-seq: RNA sequencing
H&E: hematoxylin and eosin
-Wnt: Wnt inhibition
Ctrl: control mice
TGF-β: transforming growth factor-beta
-TGF-βe: TGF-β inhibitor early
-TGF-βl: TGF-β inhibitor late
3T3: NIH-3T3
si: RNA interference
