# Supplementary material for: Dedifferentiated Schwann cell-derived TGF-β3 is essential for the neural system to promote wound healing
Source: Theranostics. 2022 Jul 18;12(12):5470–87. doi: 10.7150/thno.72317 (PMC9330527; doi:10.7150/thno.72317)
Supplement: Supplementary file 1 — Supplementary figures and tables. [file thnov12p5470s1.pdf]

Supplementary Figure S1

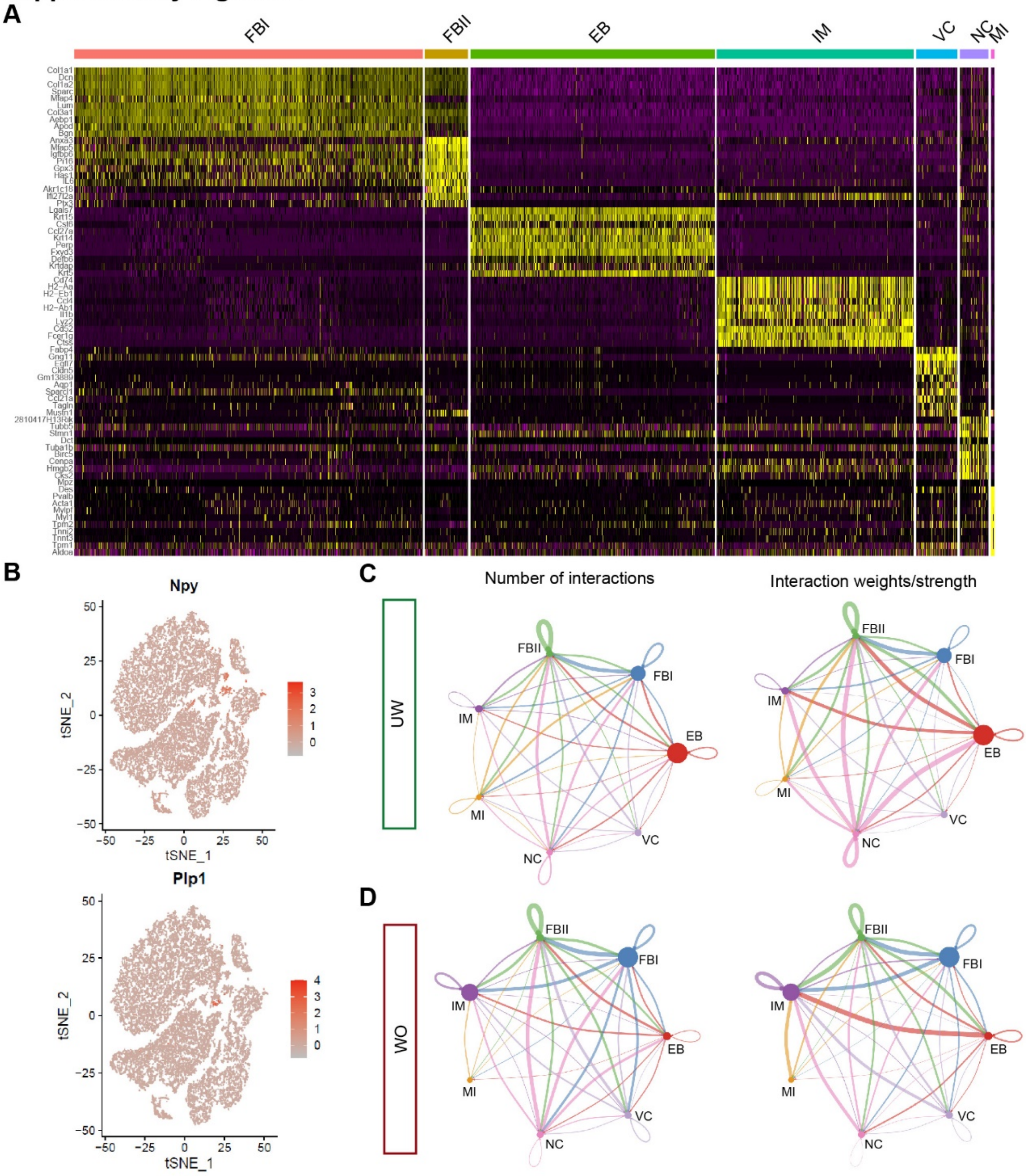

Figure S1. Identification of cell types from an integrated library of mouse skin. **(A)** Heatmap of DEGs in each cell type. The top 10 DEGs and their relative expression levels in all sequenced cells are shown. **(B)** UMAP showing expression level of *Npy* and *Plp1*. **(C, D)** Numbers (left) and relative strength (right) of significant ligand–receptor pairs among cell types in UW (C) or WO (D) skin. The edge width indicates the number or relative strength of ligand–receptor pairs. The circle sizes are proportional to the numbers of each cell type. Heatmap of the total number of putative interactions between cell types in UW skin and WO skin

Supplementary Figure S2

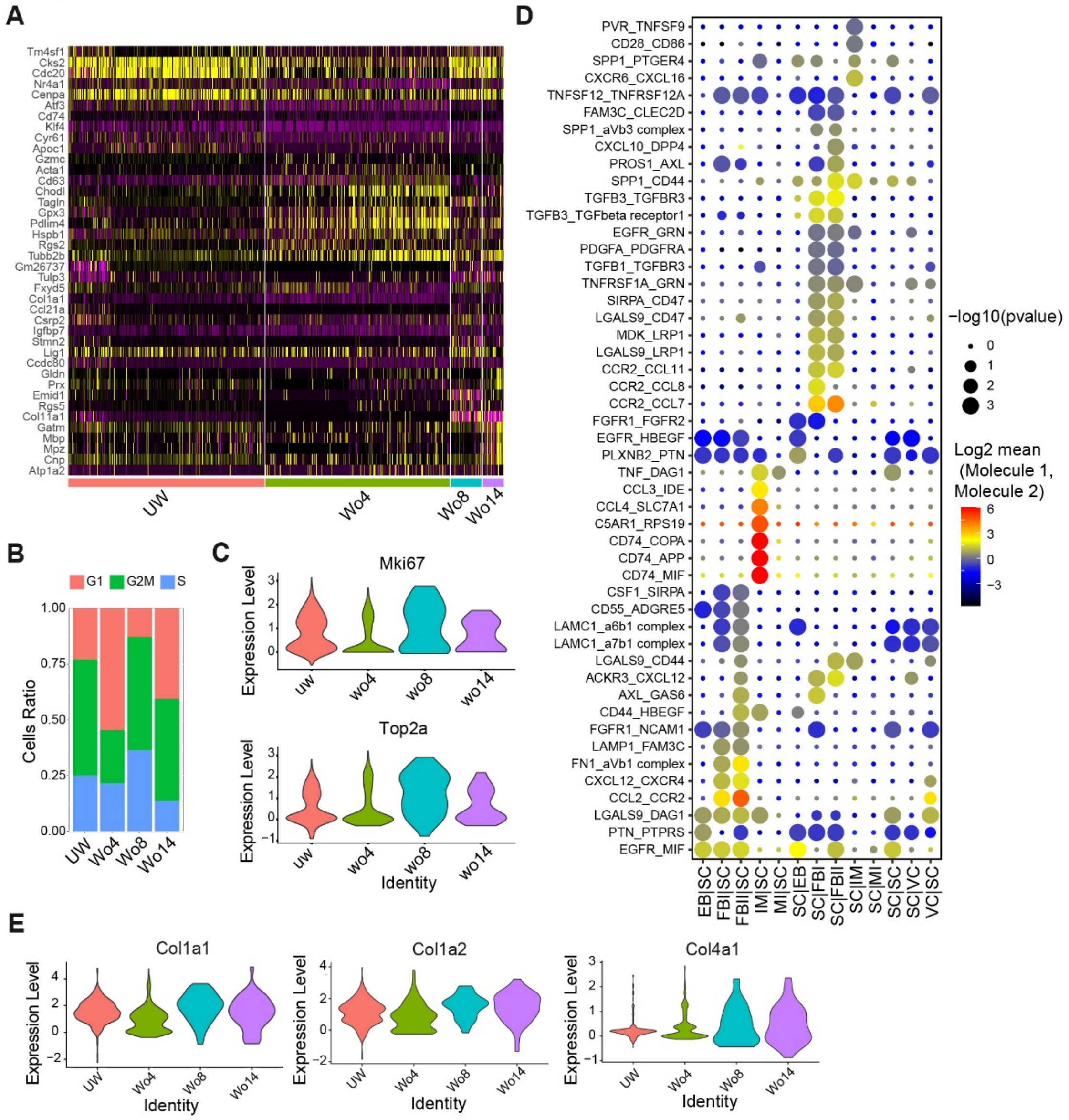

Figure S2. Subpopulations of SCs with gene expression differences.

(A) Heatmap showing the top 10 DEGs in all SCs classified by time. **(B)** Cell cycle identities of SCs at each time point. **(C)** Expression levels of *Mki67* and *Top2a* in SCs at different time points. **(D)** Dot plot overview of the significant ligand–receptor interactions between SCs and other cells at Wo4. **(E)** Expression levels of *Col1a1*, *Col1a2* and *Col4a1* in SCs at different time points.

Supplementary Figure S3

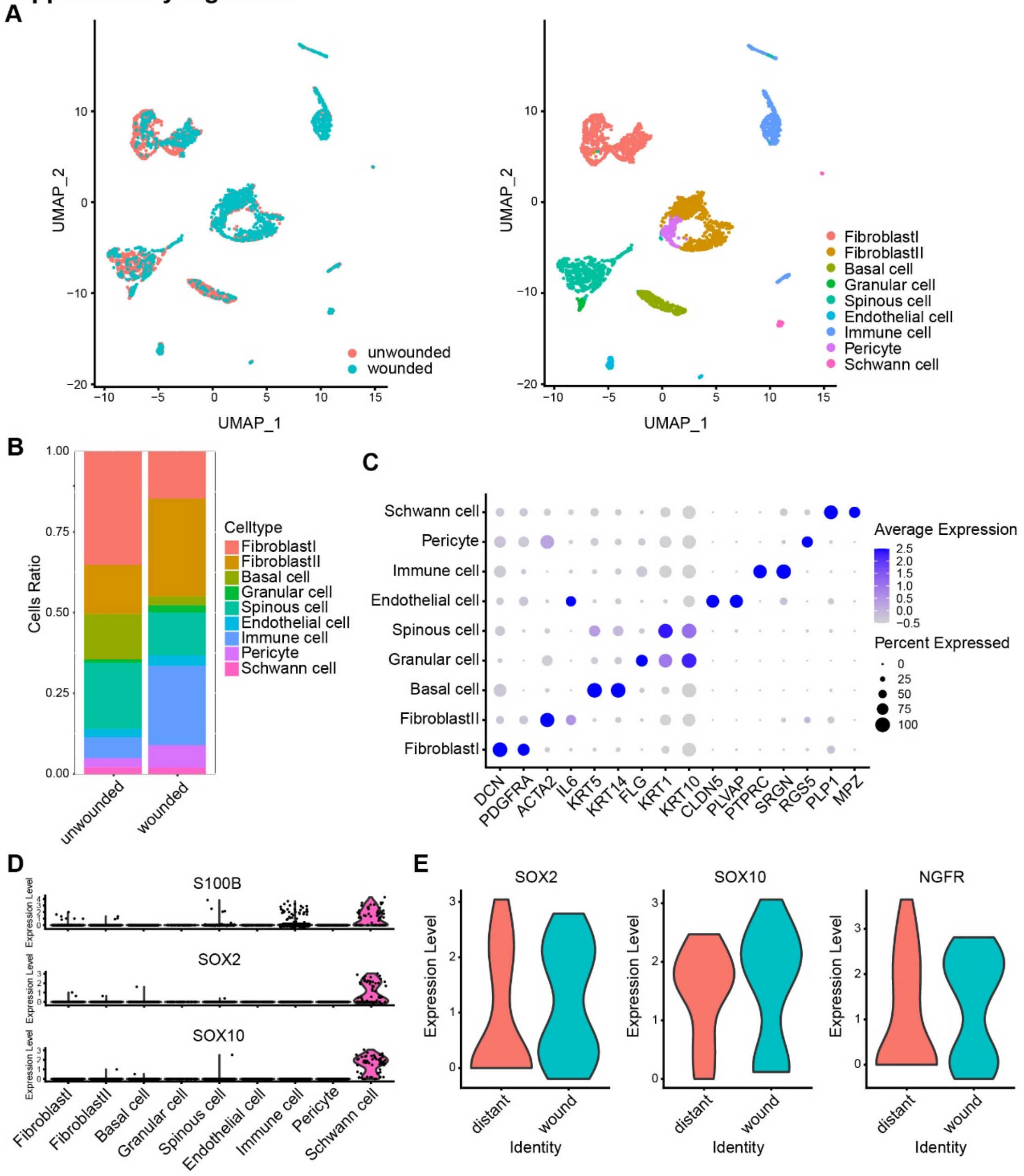

Figure S3. ScRNA analysis of chronic wounds in diabetic patients. (A) UMAP plot showing the cells from diabetic patient skin with or without injury (wounded and unwound). Raw scRNA-seq data from GSE154557. The cells are colored by source (left) or cell type (right) identity. **(B)** Bar graph showing the cell type populations in the unwounded and wounded samples. **(C)** Dot plot of signature genes expression in different cell types. **(D)** Expression level of the denoted genes in each cell type. **(E)** Expression level of the denoted genes in unwounded or wounded samples.

Supplementary Figure S4

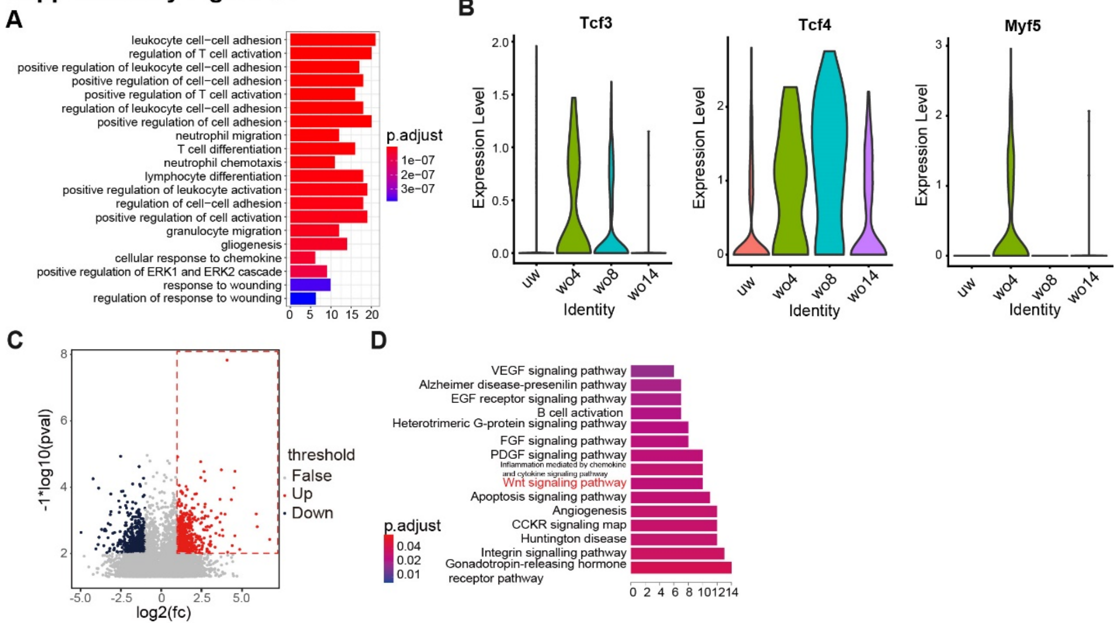

Figure S4. Genes in the Wnt signaling pathway are upregulated in dedifferentiated SCs.

(A) Enriched GO terms for the significantly upregulated genes in Wo4 SCs.

(B) Expression levels of *Tcf3*, *Tcf4* and *Myf5* in SCs at different times. **(C)** Volcano plot showing the DEGs identified by *Ballgown* (adjusted p values < 0.05 and fold changes ≥ 2) between SCs from UW and WO skin. **(D)** GO enrichment of the upregulated genes in SCs from injured skin.

Supplementary Figure S5

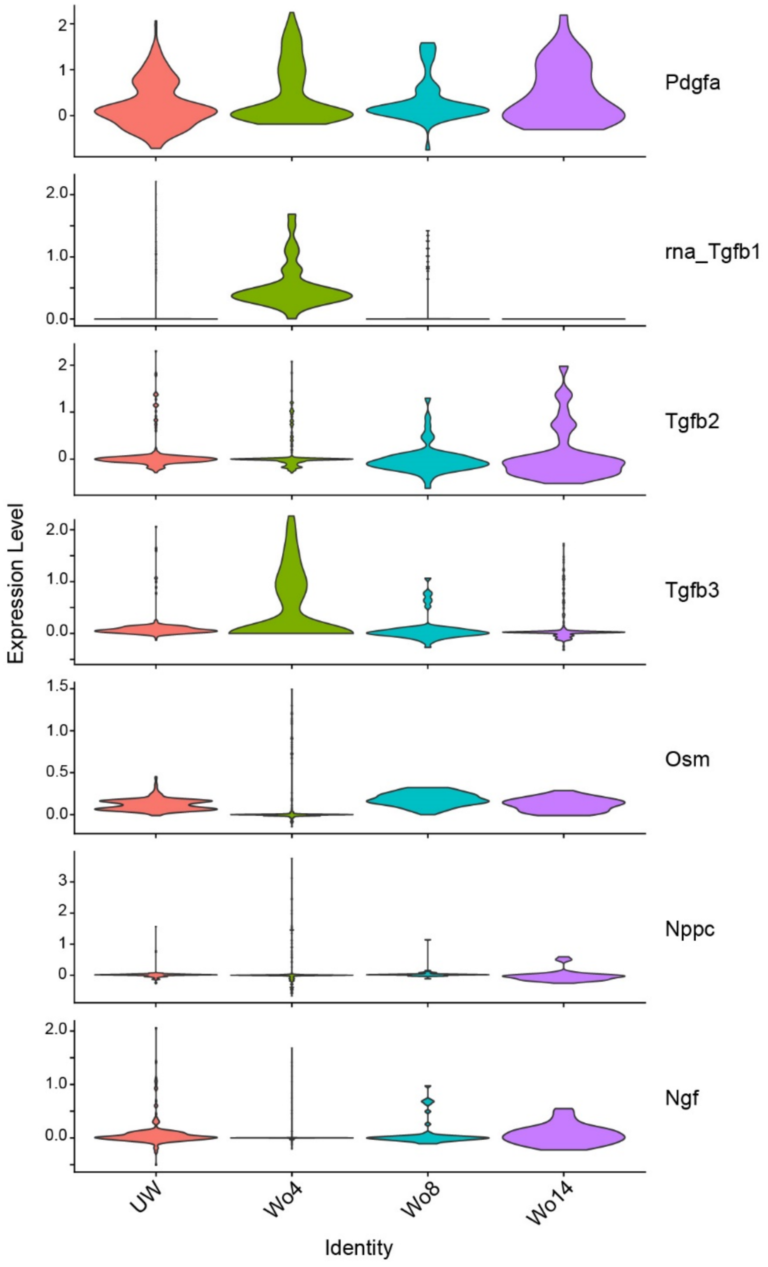

Figure S5. SCs secrete growth factors during wound healing.

Expression levels of genes encoding growth factors at each denoted time. '*Rna_Tgfb1*' means that expression level of *Tgfb1* was too low to detect in default search locations, and was determined by the RNA assay instead.

Supplementary Figure S6

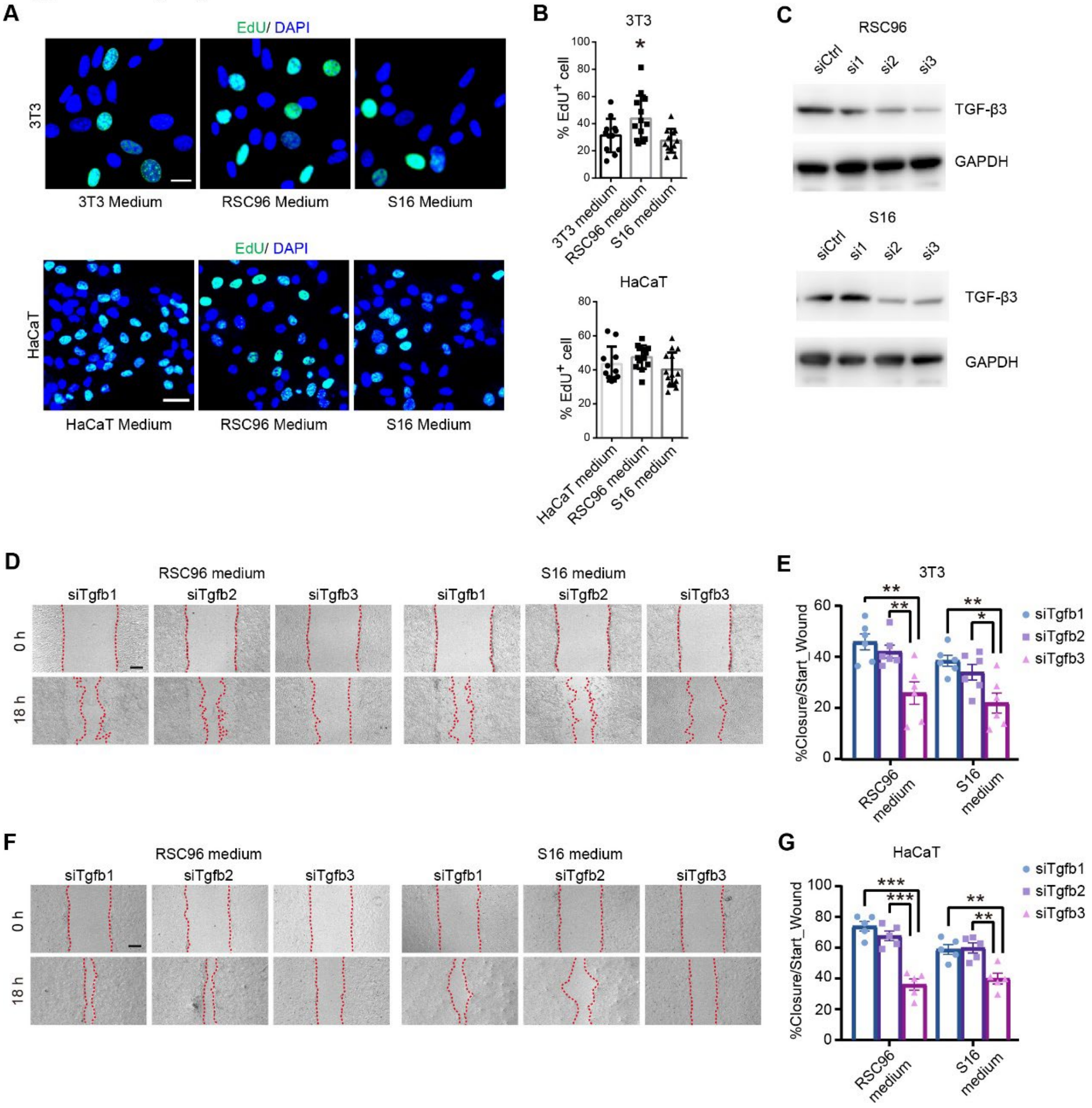

Figure S6. SCs have little effect on the proliferation of keratinocytes and fibroblasts.

(A) Immunostaining of EdU in the denoted cells with S16 or RSC96 medium treatment. The cells were incubated with EdU for 2 h to label proliferative cells. Scale bar, 20 μm. **(B)** Quantification of the percentages of EdU-positive cells among the denoted cells treated with S16 or RSC96 medium. One-way ANOVA. **(C)** Verification of the efficiency of downregulation by RNAi in 3T3 or HaCaT cells. The images are representative of three independent experiments. **(D)** In vitro wound-healing assays of 3T3 cells cultured in medium from RSC96 or S16 cells after *Tgfb1*, *Tgfb2* or *Tgfb3* RNAi (siTgfb1, siTgfb2 or siTgfb3) treatment. Scale bars: 100 μm. **(E)** Quantification

of wound closure of 3T3 cells under different conditions. **(F)** In vitro wound-healing assays of HaCaT cells cultured under the indicated conditions. Scale bars: 100 μm. **(G)** Quantification of wound closure in HaCaT cells under the indicated conditions. The data are presented as the mean ± SEM, *P<0.05, **P < 0.01, ***P < 0.001.

Supplementary Figure S7

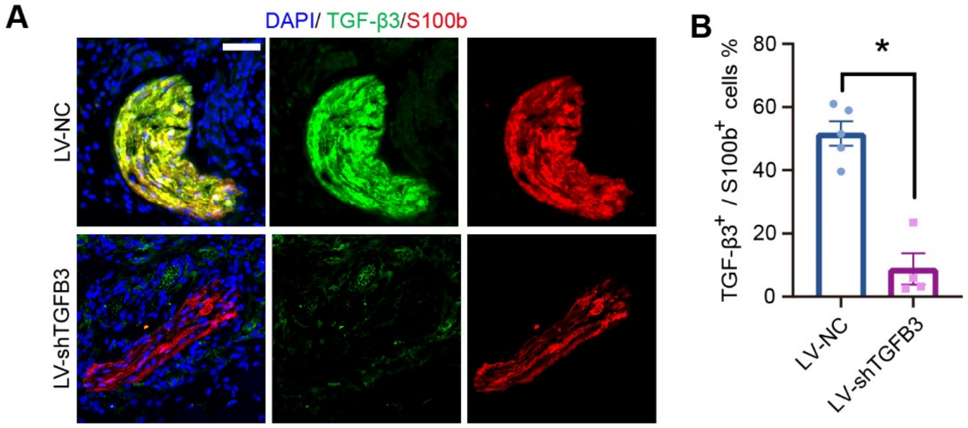

Figure S7. TGF-β3 knockdown by lentiviral injection. (A) Representative TGF-β3 immunostaining of wounds from mice injected with control (LV-NC) or Tgfb3 RNAi (LV-shTgfb3) virus at D7. Scale bar, 50 μm. **(B)** Quantification of the TGF-b3$^+$S100b$^+$ cells in LV-NC and LV-shTgfb3 wounds at D7. Unpaired t test. The data are presented as the mean ± SEM, *P<0.05, **P < 0.01, ***P < 0.001.

Supplementary Table S1. Top10 DEGs of each cell type in integrated library.

Supplementary Table S2. Enriched GO term in FBs and EBs at Wo4.

Supplementary Table S1. Top10 DEGs of each cell type in integrated library.

Cell type	p_val	avg_log2FC	pct.1	pct.2	p_val_adj	gene
FBI	0	4.156347842	0.999	0.905	0	Col1a1
FBI	0	4.043112872	0.999	0.935	0	Dcn
FBI	0	3.97087383	0.999	0.912	0	Col1a2
FBI	0	3.773622345	1	0.914	0	Sparc
FBI	0	3.6871193	0.95	0.77	0	Mfap4
FBI	0	3.418180691	0.985	0.782	0	Lum
FBI	0	3.402281852	0.999	0.924	0	Col3a1
FBI	0	3.16549914	0.993	0.669	0	Aebp1
FBI	0	3.113064944	0.979	0.81	0	Apod
FBI	0	3.045298858	0.994	0.73	0	Bgn
FBII	0	2.87518034	0.977	0.616	0	Anxa3
FBII	0	2.778879401	0.988	0.734	0	Mfap5
FBII	0	2.597960369	0.995	0.794	0	Igfbp6
FBII	0	2.578777586	0.977	0.744	0	Pi16
FBII	0	2.512322436	0.995	0.732	0	Gpx3
FBII	0	2.359922892	0.937	0.707	0	Has1
FBII	0	2.240110717	0.983	0.814	0	IL6
FBII	0	2.219787748	0.881	0.543	0	Akr1c18
FBII	0	2.217525004	0.99	0.866	0	Ifi27l2a
FBII	8.18E-293	2.436194504	0.836	0.599	1.64E-289	Ptx3
EB	0	5.535975783	0.999	0.609	0	Lgals7
EB	0	4.651335674	0.991	0.567	0	Krt15
EB	0	4.179023585	0.835	0.568	0	Cst6
EB	0	4.136573861	0.962	0.501	0	Ccl27a
EB	0	4.092318775	0.982	0.505	0	Krt14
EB	0	3.885813811	0.997	0.488	0	Perp
EB	0	3.861582444	0.996	0.535	0	Fxyd3
EB	0	3.810233448	0.899	0.56	0	Defb6
EB	0	3.747865129	0.672	0.471	0	Krtdap
EB	0	3.745093296	0.97	0.447	0	Krt5
IM	0	5.713785837	0.962	0.948	0	Cd74
IM	0	4.970252146	0.927	0.938	0	H2-Aa
IM	0	4.793073116	0.937	0.94	0	H2-Eb1
IM	0	4.728762043	0.926	0.953	0	Ccl4
IM	0	4.577708034	0.948	0.961	0	H2-Ab1
IM	0	4.307171654	0.931	0.933	0	Il1b
IM	0	4.159877763	0.761	0.741	0	Lyz2
IM	0	3.673460775	0.987	0.855	0	Cd52
IM	0	3.49654016	0.939	0.867	0	Fcer1g
IM	0	3.464572454	0.937	0.78	0	Ctss
VC	0	4.893025896	0.89	0.616	0	Fabp4
VC	0	3.124881947	0.972	0.676	0	Gng11
VC	8.84E-290	2.856720321	0.779	0.386	1.77E-286	Egfl7
VC	4.65E-251	4.137967542	0.742	0.335	9.30E-248	Cldn5
VC	5.71E-224	3.614284436	0.804	0.543	1.14E-220	Gm13889
VC	7.52E-220	2.648098779	0.812	0.529	1.50E-216	Aqp1
VC	1.68E-144	2.927174277	0.761	0.66	3.35E-141	Sparcl1
VC	1.53E-108	6.307335136	0.64	0.402	3.07E-105	Ccl21a
VC	2.47E-88	3.520475993	0.674	0.451	4.94E-85	Tagln
VC	7.25E-62	2.655080212	0.655	0.566	1.45E-58	Mustn1
NC	2.86E-167	1.813927761	0.789	0.515	5.73E-164	2810417H13Rik
NC	2.47E-154	1.9093649	0.923	0.813	4.94E-151	Tubb5
NC	6.44E-153	2.249448236	0.772	0.582	1.29E-149	Stmn1
NC	2.82E-149	2.674339593	0.721	0.526	5.64E-146	Dct
NC	1.19E-146	1.797911653	0.925	0.807	2.39E-143	Tuba1b
NC	8.14E-136	1.525221283	0.751	0.533	1.63E-132	Birc5
NC	9.92E-134	1.389860856	0.805	0.618	1.98E-130	Cenpa

NC	1.57E-125	2.449749123	0.824	0.717	3.15E-122	Hmgb2
NC	1.59E-92	1.498344782	0.749	0.613	3.18E-89	Cks2
NC	2.56E-06	2.22322135	0.407	0.358	0.005115738	Mpz
MI	7.02E-79	5.627684464	1	0.564	1.40E-75	Des
MI	1.08E-78	7.949359163	1	0.701	2.16E-75	Pvalb
MI	1.11E-78	8.497561379	1	0.749	2.22E-75	Acta1
MI	1.13E-78	7.716565279	1	0.709	2.27E-75	Mylpf
MI	1.57E-78	7.339458846	1	0.689	3.14E-75	Myl1
MI	2.55E-78	5.509377654	1	0.668	5.10E-75	Tpm2
MI	5.11E-77	6.67994533	1	0.693	1.02E-73	Tnni2
MI	2.03E-76	6.807964582	0.992	0.681	4.05E-73	Tnnt3
MI	2.06E-76	5.898061356	0.992	0.69	4.12E-73	Tpm1
MI	2.29E-75	5.026826691	1	0.91	4.57E-72	Aldoa

Supplementary Table S2. Enriched GO term in FBs and EBs at Wo4.

Go analysis of high expression genes in EB

source	term_name	term_id	adjusted_p_value	negative_log10_of_adjusted_p_value	term_size	query_size	intersection_size	effective_domain_size
GO:BP	response to stress	GO:0006950	7.25E-10	9.139575265	3817	103	51	21118
GO:BP	response to external stimulus	GO:0009605	6.05E-09	8.218106307	2912	103	43	21118
GO:BP	epithelium development	GO:0060429	1.56E-08	7.807390561	1171	103	27	21118
GO:BP	skin development	GO:0043588	1.62E-07	6.789181495	277	103	14	21118
GO:BP	locomotion	GO:0040011	8.78E-07	6.056461016	1839	103	31	21118
GO:BP	cell motility	GO:0048870	1.52655E-06	5.816290025	1653	103	29	21118
GO:BP	cell migration	GO:0016477	1.65578E-05	4.780998245	1491	103	26	21118
GO:BP	response to wounding	GO:0009611	0.000454891	3.342092932	517	103	14	21118
REAC	Keratinization	REAC:R-MMU-6805567	8.819E-05	4.054580884	166	63	10	8631
WP	Oxidative Stress	WP:WP412	0.009408631	2.026473555	28	50	4	4526

Go analysis of high expression genes in FBI

source	term_name	term_id	adjusted_p_value	negative_log10_of_adjusted_p_value	term_size	query_size	intersection_size	effective_domain_size
GO:BP	cellular response to chemical stimulus	GO:0070887	3.20E-11	10.49531154	3097	77	40	21118
GO:BP	response to external stimulus	GO:0009605	1.65E-10	9.781521749	2912	77	38	21118
GO:BP	tissue development	GO:0009888	4.16E-10	9.380773565	1923	77	31	21118
GO:BP	response to oxygen-containing compound	GO:1901700	4.58E-09	8.339374896	1820	77	29	21118
GO:BP	response to stress	GO:0006950	6.51E-09	8.186329315	3817	77	41	21118
GO:BP	response to wounding	GO:0009611	8.79E-08	7.056145889	517	77	16	21118
GO:BP	wound healing	GO:0042060	2.11125E-06	5.675459495	380	77	13	21118
GO:BP	cell motility	GO:0048870	4.30153E-06	5.366376919	1653	77	24	21118
GO:BP	cell differentiation	GO:0030154	1.04988E-05	4.978859818	4378	77	39	21118
GO:BP	regulation of cell adhesion	GO:0030155	1.06909E-05	4.970987006	720	77	16	21118

Go analysis of high expression genes in FBII

source	term_name	term_id	adjusted_p_value	negative_log10_of_adjusted_p_value	term_size	query_size	intersection_size	effective_domain_size
GO:BP	response to stress	GO:0006950	4.03E-22	21.39513369	3817	99	65	21118
GO:BP	response to external stimulus	GO:0009605	4.40E-14	13.35691696	2912	99	49	21118
GO:BP	cell differentiation	GO:0030154	8.10E-14	13.0916564	4378	99	59	21118
GO:BP	cell migration	GO:0016477	8.77E-14	13.05675716	1491	99	36	21118
GO:BP	locomotion	GO:0040011	9.65E-12	11.01547247	1839	99	37	21118
GO:BP	regulation of response to stress	GO:0080134	1.38E-11	10.85942431	1254	99	31	21118
GO:BP	innate immune response	GO:0045087	2.77E-09	8.557585359	866	99	24	21118
KEGG	IL-17 signaling pathway	KEGG:04657	5.59E-12	11.25297063	91	65	13	8790
KEGG	TNF signaling pathway	KEGG:04668	3.94E-08	7.404891897	113	65	11	8790
WP	TGF-beta Signaling Pathway	WP:WP366	0.041613323	1.380767602	133	75	7	7562
